# Connecting the Dots between PubMed Abstracts

**DOI:** 10.1371/journal.pone.0029509

**Published:** 2012-01-03

**Authors:** M. Shahriar Hossain, Joseph Gresock, Yvette Edmonds, Richard Helm, Malcolm Potts, Naren Ramakrishnan

**Affiliations:** 1 Department of Computer Science, Virginia Tech, Blacksburg, Virginia, United States of America; 2 Department of Biochemistry, Virginia Tech, Blacksburg, Virginia, United States of America; 3 Department of Biological and Environmental Sciences, Qatar University, Doha, Qatar; The Centre for Research and Technology, Hellas, Greece

## Abstract

**Background:**

There are now a multitude of articles published in a diversity of journals providing information about genes, proteins, pathways, and diseases. Each article investigates subsets of a biological process, but to gain insight into the functioning of a system as a whole, we must integrate information from multiple publications. Particularly, unraveling relationships between extra-cellular inputs and downstream molecular response mechanisms requires integrating conclusions from diverse publications.

**Methodology:**

We present an automated approach to biological knowledge discovery from PubMed abstracts, suitable for “connecting the dots” across the literature. We describe a storytelling algorithm that, given a start and end publication, typically with little or no overlap in content, identifies a chain of intermediate publications from one to the other, such that neighboring publications have significant content similarity. The quality of discovered stories is measured using local criteria such as the size of supporting neighborhoods for each link and the strength of individual links connecting publications, as well as global metrics of dispersion. To ensure that the story stays coherent as it meanders from one publication to another, we demonstrate the design of novel coherence and overlap filters for use as post-processing steps.

**Conclusions:**

We demonstrate the application of our storytelling algorithm to three case studies: i) a many-one study exploring relationships between multiple cellular inputs and a molecule responsible for cell-fate decisions, ii) a many-many study exploring the relationships between multiple cytokines and multiple downstream transcription factors, and iii) a one-to-one study to showcase the ability to recover a cancer related association, viz. the Warburg effect, from past literature. The storytelling pipeline helps narrow down a scientist's focus from several hundreds of thousands of relevant documents to only around a hundred stories. We argue that our approach can serve as a valuable discovery aid for hypothesis generation and connection exploration in large unstructured biological knowledge bases.

## Introduction

The use of high-throughput data screens in biology [1], [2] has resulted in an ever-growing stream of information about genes, proteins, pathways, and diseases. Due to the increase in the number of papers published based on these large-scale studies [3], [4], there is growing acknowledgment of the need to automatically integrate information across multiple publications [5]–[9]. Such a task is key to gaining insight into the functioning of biological systems as a whole. In particular, such integration can help formulate biological hypotheses in areas that are still too expensive or too tentative to study by traditional experimental methods. The automatic formulation of new biological hypotheses or discovery of hidden connections from published literature is thus a very attractive but non-trivial task.

There are two broad classes of prior work in this area. The first focuses on mining the literature to generate targeted hypotheses about associations and interactions between genes, proteins, or diseases. Yu and Agichtein [10], for instance, observe that genes and proteins are often associated with multiple names, and use supervised and unsupervised machine learning techniques to extract synonymous gene and protein terms from biological literature. There is significant work in automatic construction of biological interaction networks [11]–[14] by mining textual sources, e.g., PASTA [15], Chilibot [16], PubGene [17], ONDEX [18], iHOP [19], and BioGraph [20]. Some of these tools are aimed at generating hypotheses (e.g., [16], [19], [20]) but others are more simply intended to discover interactions between biological entities. There has been a heavy adaptation of natural language processing (NLP) techniques toward biological knowledge discovery. Rindflesch et al. [21] and Hatzivassiloglou and Weng [22], for instance mine interaction verbs from biomedical text; more sophisticated entity recognition and dependency parsing tools have also been used [7], [23]–[31]. Statistical NLP techniques, e.g., co-occurrence and concept-based approaches [32]–[37], kernel-based techniques [38], [39], clustering-based methods [40], [41] are slowly becoming a mainstay in this field.

The second class of work, and more relevant for our purposes, constitutes open-ended discovery tools and systems that aim to relate published literature and automatically generate hypotheses. Swanson is an early pioneer in this space with his work on disjoint science literature [42]. He proposes the so-called complementary but disjoint (CBD) strategy wherein two arguments may exist separately that when considered together lead to new insights, but the papers exhibiting these two arguments appear unaware of each other. In two different works [43], [44], Swanson demonstrated examples of important but unnoticed connections hidden in isolated biomedical literature. For instance, he extracts a connection from migraine to magnesium by relating through inflammation research. The early implementations of the CBD strategy were co-occurrence based and intended to be interactive, so that each step of the story was discovered by user selection from a set of system-provided choices. Wren et al. [45], Srinivasan and Libbus [46], and Eijk et al. [47] extend and improve this discovery approach of Swanson by proposing scoring functions for automated exploration of links in story construction. Srinivasan and Libbus [46], in particular, use a weighted vector-space model of MeSH terms as the underlying representation rather than the original documents. They show how several diseases of the retina and other disorders can be related to a dietary supplement (Curcumin Longa) popular in Asia. All these papers describe methodologies to generate hypotheses that have not been reported in a single paper before and that potentially lead to new directions for experimental validation.

In this paper, we demonstrate a storytelling algorithm that can connect the dots between PubMed abstracts. The focus on abstracts rather than full-length articles is due to the broader availability of free abstracts and the more sophisticated machine learning models we can apply at the abstract level. Given a start and end publication, typically with little or no overlap in content, our algorithm identifies a chain of intermediate publications from one to the other, such that neighboring publications have significant content similarity.

Our work extends the above projects in several ways. First, in contrast to Swanson [42], Srinivasan and Libbus [46], and similar works, we propose the discovery of stories involving longer chains of documents than two/three documents (as these works focus on). This enables us to connect a broader range of literature and thus generate a richer variety of hypotheses. Second, to ensure that longer chains do not lose coherence, we propose the design of neighborhood constraints to enforce better organization of surrounding evidence for each link of the story. This enables the biologist to interactively guide the search for stories to suit desired quality goals. Third, we adopt the CBD structure to generate new hypotheses that might be missed if we imposed all-pairs commonality constraints. Fourth, in contrast to Wren et al. [45] and Eijk et al. [47], we demonstrate novel algorithms for incrementally constructing stories from start to end, without materializing the complete similarity graph (which can be prohibitively expensive for the collection sizes considered here). Finally, we propose the design of novel filters to judge context overlap and coherence among the story links, leading to a powerful set of techniques for generating biologically interpretable and summarizable stories.

### Example Story

As an example of the capabilities envisaged here, we explore the similarities in adaptation to metabolic arrest (quiescence) between yeast and complex eukaryotes such as mice. We begin with a paper that focuses on yeast:

K.N. Maclean, M. Janosík, E. Kraus, V. Kozich, R.H. Allen, B.K. Raab, J.P. Kraus, Cystathionine beta-synthase is coordinately regulated with proliferation through a redox-sensitive mechanism in cultured human cells and *Saccharomyces cerevisiae*, *J Cell Physiol*, Vol. 192 No. 1, pages 81–92, Jul 2002.

and aim to connect it to a paper about mice:

E. Blackstone, M. Morrison, and M.B. Roth, H_2_S induces a suspended animation-like state in mice, Science, Vol. 308, No. 5721, page 518, Apr 2005.

While such an inter-species correlation may appear initially to be far-fetched, metabolic arrest is a process inherent to all organisms. One would thus assume that there are process similarities, in this case sulfur metabolism, across all forms of life. Testing such relationships may provide new insights into this important process. We demonstrate a manually conceptualized story here to motivate our algorithmic approach.

By studying the abstract of the first paper, we observe the mention of CBS (cystathionine-beta synthase) domains, which are small intracellular modules, mostly found in two or four copies within a protein, that occur in several different proteins in all kingdoms of life. We might begin by looking for other publications that discuss CBS. Using this idea, we can reach:

L. Schmitt and R. Tampe, Structure and mechanism of ABC transporters, Current Opinion in Structural Biology, Vol. 14, No. 4, pages 426–431, Aug 2004.

From this paper, we learn that CBS domains are found in glycine betaine transport proteins that mediate osmotic adjustment in cells. Following this thread and investigating CBS domains further leads to the paper:

J.W. Scott, S.A. Hawley, K.A. Green, M. Anis, G. Stewart, G.A. Scullion, D.G. Norman, and D.G. Hardie, CBS domains form energy-sensing modules whose binding of adenosine ligands is disrupted by disease mutations, Journal of Clinical Investigation, Vol. 113, No. 2, pages 182–184, Jan 2004.

This publication reveals the nature of interactions found in molecular complexes involving CBS domains. At this point, we shift the emphasis from CBS to the function of ligands mentioned in this paper, to reach:

C. Tang, X. Li and J. Du, Hydrogen sulfide as a new endogenous gaseous transmitter in the cardiovascular system, Current Vascular Pharmacology, Vol. 4, No. 1, pages 17–22, Jan 2006.

This paper indicates that in humans, hydrogen sulphide (H_2_S) is produced endogenously in mammalian tissues from L-cysteine metabolism mainly by three enzymes, one of which is CBS. In addition, H_2_S may not only function as a neuromodulator in the central nervous system but also serve to relax gastrointestinal smooth muscles. We now look for connections involving H_2_S, leading to our intended target, a publication about mice:

E. Blackstone, M. Morrison, and M.B. Roth, H_2_S induces a suspended animation-like state in mice, Science, Vol. 308, No. 5721, page 518, Apr 2005.

The story thus mined, through the sequence of intermediaries, provides a continuous chain of reasoning about metabolic arrest across organisms of diverse complexity. It is important to note that the story proceeds through sulfur metabolism, a feature that we had identified in a previous study as an integral part of the desiccation and recovery process of baker's yeast, *Saccharomyces cerevisiae* [48]. This application of storytelling suggests that laboratory-based efforts aimed at understanding the role of sulfur metabolism in metabolic arrest is an area worth exploring in detail.

Stories as depicted above reveal multiple forms of insights. Since intermediaries must conform to a priori knowledge, we can think of storytelling as a carefully argued process of removing and adding participants, not unlike a real story. Furthermore, similar to books such as Burke's *The Knowledge Web* [49], the network of stories underlying a domain reinforces interconnections between ideas rather than strict sequential and historical progression of discoveries. In particular, storytelling reveals if certain papers (and hence, concepts) have greater propensity for participating in some stories more than others. Such insights have great explanatory power and help formulate hypotheses for situating new data in the context of well-understood processes.

It is important to recognize that stories yield a sequential chain of documents from a starting point to an ending point unlike results from search engines (e.g, Google) which are a disconnected set of documents for a single query. To mimic a storytelling capability using a search engine would require the biologist to issue two separate queries, manually inspect results from each query, and issue further queries in an attempt to reach one set of documents from another. Needless to say, this process is not likely to be very effective. A more tractable approach is to use PubMed's “related research” links to explore the underlying network of documents but even this approach will be fraught with dead-ends and back-and-forth exploration of document similarities. Our goal is to design an automated storytelling algorithm that can significantly lower the barrier for biologists to explore long connections between desired start and end points (documents).

Formally, storytelling can be viewed as a generalization of redescription finding. Redescriptions are ways to re-state information from one data vocabulary into another, typically to aid insight into commonalities and distinctions across vocabularies. The redescription finding problem was introduced in [50] with additional theoretical foundations described in [51]–[53]. The storytelling problem was formulated in [54], [55] but the algorithmic innovations were geared toward Boolean set vocabularies. Here, we describe a comprehensive approach to storytelling in document collections, paying particular attention to cohesion and interpretability of stories.

### Motivating Problems

#### Organismal aging

Our research group is interested in the molecular mechanisms underlying organismal aging, i.e., the ability/inability of cells to have a controlled yet extended lifespan. We recently demonstrated that the supplementation of nicotinamide to the growth medium of primary fibroblasts provided a lifespan extension [56], and this lifespan extension was subsequently reported by others [57], [58]. In order to generate testable hypothesis to understand this lifespan extension at the molecular level, we need to elucidate relationships between cellular inputs (such as nicotinamide) and outputs (cell fate decisions such as lifespan extension).

Nicotinamide, a component of nicotinamide adenine dinucleotide (NAD^+^), is usually considered a cofactor in redox reactions, but is also known to be a substrate in protein deacetylation and ‘ADP-ribosylation’ reactions. Considering the equilibrium nature of biochemical reactions, it is not surprising that high levels of nicotinamide inhibit these reactions. ADP-ribosylation is an enzyme-mediated reaction whereby NAD^+^ is converted to ADP-ribose, releasing nicotinamide. The ADP-ribose group can then be attached to a protein post-translationally to form chains of ADP-ribose polymers, or to form ADP-ribose polymers free of protein. ADP-ribosylation is associated with the DNA damage response, cell death processes, as well as chromatin remodeling [59]. PARP-1, the enzyme that generates ADP-ribose chains, is one of the most abundant proteins in the nucleus of human cells and PARP-1 inhibition is presently an active line of pharmaceutical research.

Building upon our nicotinamide supplementation work, we performed a series of transcriptional profile experiments (unpublished data) to determine if there were any genes that were significantly up- or down-regulated due to the nicotinamide addition. While several genes were identified through this work, there were no obvious links in the literature, especially to ADP-ribosylation inhibition. We also evaluated primary fibroblasts for their ability to metabolically arrest for extended periods (a form of lifespan extension), and several cytokines were implicated in this response [60]. Again we sought to identify links between these extracellular molecules and ADP-ribosylation.

To computationally model this problem, we cast it as one of exploring combinatorial relationships in the literature between a set of 18 input extracellular molecules (see Table 1) and one output, i.e., (poly)ADP-ribose. How do signaling cascades and cellular responses to these molecules interact? Are there overlapping pathways or largely independent cascades of input-output relationships? For each molecule, we pick a representative document discussing it, attempt to create a story to a document discussing ADP-ribos(ylation), and study the discovered stories to understand the chains of connections.

**Table 1:** Case Study 1.

Type	Molecule	Function/Comment
**Input**	CD38	ADP-ribosylcyclase 1, modulator of NAD levels
	CXCL1	Growth-regulated protein alpha precursor, chemokine
	IL-8	Interleukin-8, chemotactic cytokine (CXCL8), inflammatory response
	IL-1β	Interleukin-1beta, cytokine, inflammatory response
	IL-6	Interleukin-6, cytokine, multiple functions
	IL-13	Interleukin-13, cytokine, multiple functions
	IL-24	Interleukin-24, cytokine, antiproliferative properties
	MCP-1 and 2	Monocyte chemotactic proteins; mitogenic, chemotactic, and inflammatory activity
	IFN-γ	Interferon-gamma; antiviral, antiproliferative, and immunoregulatory functions
	Nicotinamide (NAM)	Lifespan extension in primary fibroblasts
	STC-1	Stanniocalcin-1; phosphate regulator, up-regulated in nicotinamide microarray experiments
	IGF-1	Insulin-like growth factor 1, downregulated in NAM supplementation studies
	SFRP-1	Secreted frizzled-related protein 1, function unknown, upregulated in NAM supplementation studies
	Matrix metalloproteinase	Extracellular proteinases (MMP), involved in extracellular matrix degradation
	MMP 3	MMP, downregulated in NAM supplementation studies, implicated in wound repair, atherosclerosis, and tumor initiation
	MMP 12	Extracellular proteinase, downregulated in NAM supplementation
	Serpin B-2/PAI-2	Degrades elastin, serine or cysteine proteinase inhibitor
**Output**	Poly(ADP-ribose)	DNA repair and programmed cell death

Storytelling inputs and outputs used to explore combinatorial relationships. All input molecules listed are extracellular except CD38, which is bound to the outer membrane.

#### Interactions between transcription factors and cytokines

A second case study presented herein involves connections between *multiple* cytokines and *multiple* transcription factors. Tumor Necrosis Factor-alpha (TNF-alpha), Interleukin-1beta (IL-1beta), and Interleukin-8 (IL-8) are all cytokines that activate similar transcription factors, such as Activator Protein-1 (AP-1) members and Nuclear Factor-kappa B (NF-kB). Interferon-gamma (IFN-gamma) activates some similar transcription factors but also activates different signaling pathways leading to alternate transcription factors. In addition, AP-1 family members (such as c-jun, c-fos, JunD, ATF2, and ATF3) have been shown to be regulated by NF-kB and bind to their promoters. These members act as transcription factors in dimers, so pairs such as c-jun and c-fos bind together to act as a transcription factor. Also of note is that the aforementioned cytokines not only regulate the activation of these transcription factors, but their expression is also regulated by the same transcription factors. A complex web of interactors and pathways is present in mammalian systems, with aberrant signaling associated with many disease states. Can our storytelling algorithm be used to evaluate how different cytokines interact with various transcription factors? We model this problem similarly to the previous example, this time setting the 4 cytokines to be the input keywords and 10 transcription factors to be the output keywords in a combinatorial relationship study (see Table 2).

**Table 2:** Case Study 2.

Type	Molecule	Function/Comment
**Input**	TNF-α	Tumor Necrosis Factor-alpha, involved in systemic inflammation, causes apoptic cell death
	IL-8	Interleukin-8, cytokine, inflammatory response
	IL-1β	Interleukin-1beta, cytokine, inflammatory response
	IFN-γ	Interferon-gamma; antiviral, antiproliferative, and immunoregulatory functions
**Output**	NF-*κ*B	Nuclear Factor-kappa B, activated in cytosol, enters nucleus to increase transcription of other transcription factors
	CREB	cAMP Responsive Element Binding Protein, cooperates with NF-kappa B and STAT1
	c-jun	AP-1 family member, activated by phosphorylation by JNK (a MAPK)
	c-fos	AP-1 family member, stabilized by ERK (a MAPK), can be phosphorylated by JNK
	JunD	AP-1 family member, phosphorylated by JNK and potentiates its activity
	ATF2	Activating Transcription Factor-2, AP-1 family member, phosphorylated by JNK and potentiates its activity
	ATF3	Activating Transcription Factor-3, AP-1 family member, possibly regulated by all above cytokines
	STAT1	Signal Transducer and Activator Transcription 1, activated by IFN-gamma Tyrosine Kinase Receptor, mediates transcription in nucleus
	STAT2	Signal Transducer and Activator Transcription 2, activated by IFN-gamma Tyrosine Kinase Receptor, mediates transcription in nucleus
	BRCA1	Breast Cancer 1, activated by IFN-gamma, activates upregulation of STAT1 and STAT2

Four storytelling inputs and ten outputs used to explore combinatorial relationships between cytokines (inputs) and transcription factors (outputs).

#### Warburg Effect

In our final case study, we study the relationships between glycolysis and cancer. Cancer cells multiply at a faster rate than normal somatic human cells as the restrictions associated cell division have been removed. In order to support rapid growth rates, cancer cells metabolize glucose via aerobic glycolysis, a process where both the glycolytic and oxidative phosphorylation pathways are active, but more carbon is shuttled through glycolysis than normal tissues. Originally discovered over 80 years ago, this phenomenon is termed the Warburg Effect [61]. The increased conversion of glucose to lactate leads to glucose consumption rates nine times higher than normal tissues, with lactate levels increased by up to a factor of seven. Oxygen consumption rates between the two cell types are similar indicating that cancer cells still have ongoing tricarboxylic acid (TCA) cycle activity and oxidative phosphorylation. While these phenomena have been linked to hypoxia-inducible factors, loss of tumor suppressors and activation of oncogenes, the relationships between glycolysis, lactate production, the TCA cycle, oxidative phosphorylation and cancer growth are not fully established.

Two papers appeared in 2008 [62], [63] that supported the concept [64] that the Warburg effect could be explained in part by an isoform of pyruvate kinase, PKM2. This protein was generally considered to be present in high levels in fetal and cancer tissues, two cell types undergoing rapid proliferation, but low in normal cell types. The PKM2 enzyme was less effective allowing metabolites to be funneled down anabolic pathways associated with cell growth. A subsequent study by Hitosugi et al. [65], suggested that tyrosine kinase signaling regulates PKM2 and contributes to tumorigenesis and maintenance of the tumor. These results supported the PKM2 hypothesis of Mazurek *et al*. [64] that a less active phosphorylated PKM2 permits the funneling of glucose into synthesis of lipids and amino acids rather than into pyruvate. While these observations explained the increased growth rate of cancer cells, they could not explain where the high levels of lactate came from. As some tumors have increased levels of malic enzyme and glutaminase, it was hypothesized that the source of pyruvate for conversion to lactate came from glutamine metabolism [66], [67]. Glutamine can be converted to glutamate by glutaminase and glutamate can be converted to 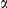-ketoglutarate (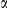-KG) by transamination or oxidation. If the source of lactate is glutamine, the path would involve conversion of glutamine to glutamate, then 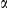-KG, which would proceed through TCA to malate. Malate can then be converted to pyruvate via malic enzyme (ME), and LDH can convert it to lactate.

As several key papers providing key insights into the metabolic needs of cancer cells appeared in 2008 and 2009 [62], [63], [65], [68], we use our storytelling algorithm to study PubMed abstracts before 2008 using the keywords pyruvate kinase and glutamine as both start and end points, in order to determine if the algorithm can identify a relationship between the two.

For ease of reference, we denote the above problems as Case Study 1, Case Study 2, and Case Study 3, respectively, in the rest of the paper.

### Research Issues in Story Generation

When attempting to mine any database with the intent of constructing a new line of thought, there are several issues that must be considered when moving from one document to another. Some of these issues manifest in many text mining applications (e.g., polysemous naming conventions, the terms Nampt = PBEF = visfatin, IL-8 = CXCL8, and numerous ways in which poly-ADP ribosylation could be written) but others are specific to storytelling. We highlight four of these specific issues to motivate the design of our storytelling algorithm.

#### Topic dilution

A typical way in which disjoint abstracts get connected through a sequence of intermediaries is as a sequence of topic dilutions followed by specializations. For instance, a story might connect two different cytokines through a cytokine review article that happens to list both. Needless to say, such stories are not particularly insightful and often suggest obvious, or “lowest common denominator” connections.

#### Contextual mismatches

The example provided earlier on sulfur metabolism related a yeast publication to a paper on mice. In general, a blind chaining of abstracts relating diverse cell lines, tissue types, and experimental settings, might not create pertinent connections. Care has to be taken, hence, in relating documents. It is easy to foresee situations where two documents discuss the same biological process but for different organisms, so that relating them might lead to an invalid or misleading connection. A successful storytelling algorithm must ensure contextual match between every pair of documents connected by links in a story.

#### Lack of Evidence

Sometimes random chaining of abstracts can result in many false positive connections and mislead a researcher by proposing weak hypotheses. The story generation mechanism should marshal evidence to support a proposed hypothesis.

#### Lack of Summarizability

For stories to provide an argued way to bridge distant concepts, they should be summarizable in succinct form using sentences from each of the abstracts. Just modeling documents as bags of words [69] might not be sufficient in this case; we require the ability to tile the mined stories using representative sentences to understand the transduction of concepts sequentially.

All of the above research issues motivate our story generation methodology. An initial chaining of publications using heuristic search techniques is then subjected to many filters that systematically prune discovered stories and help summarize the flow of hypotheses across chained abstracts.

## Methods

### Algorithmic Methodology

We use vector-space representations of documents where each document is represented as a vector of term weights. A story from document 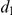 to 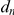 is a sequence of intermediate documents 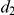, 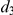, …, 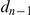 such that every neighboring pair of documents satisfies a user-specified threshold on the Soergel distance:
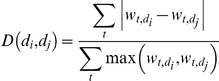
where 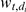 indicates the weight for term *t* of document 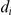. The Soergel distance is a true distance measure: it is exactly 0.0 when the documents 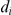 and 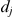 have the same term vectors, is symmetric, and obeys the triangle inequality. We also note that the Soergel distance is exactly 1.0 when the term sets of the two documents are completely disjoint.

The Soergel *distance threshold* is one handle by which we reason about the quality of stories. The normalizing nature of the Soergel distance makes it easier for the biologist to intuitively select the distance threshold parameter. Stricter thresholds on the Soergel distance give rise to longer (but potentially stronger) stories.

An alternative handle on the quality of stories aims to mimic the process by which an analyst would marshal evidence around links to create stronger stories. We define a *clique size* threshold to denote this aspect. The clique size denotes the size of the clique around each link of a story, so that larger clique sizes denote greater evidence surrounding a given link. These are not magic parameters whose values have to be tuned but are rather controls that mimic the natural process by which a biologist can tighten or strengthen the generated stories.

**Figure 1:**
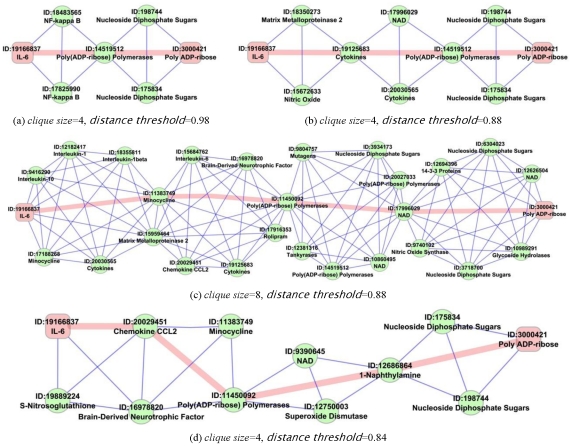
Constructing stories out of clique chains between PubMed IDs 19166837 (a document about IL-6) and 3000421 (a document about Poly ADP-ribose). Figure (a) shows a path with the least stringent requirements. As the requirements become more and more stringent in (b) and (c), the story becomes longer. Figure (d) shows a significant story from both statistical and biological viewpoints. It connects one of the inflammatory cytokines IL-6 with brain injury and Poly(ADP-ribose) Polymerase (PARP) deficiency or inhibition. The generated hypothesis suggests that PAR/PARP could impact signaling of IL-6 or other interleukins. This story is covered in detail in Table 4. Fig. 9 depicts the dispersion plots of the four stories derived from the clique chains of this figure.

In more detail, the distance threshold refers to the maximum acceptable distance between two neighboring documents in a story. Lower distance thresholds impose stricter requirements and lead to longer paths. Figure 1(a) depicts a story between a document (PubMedID: 19166837) related to IL-6 and a document (PubMedID: 3000421) related to (poly)ADP-ribose. The story becomes longer with stricter distance threshold as shown in Figure 1(b). The clique size threshold refers to the requirement of the minimum size of the clique that every pair of neighboring documents must participate in. Thus, greater clique sizes impose greater neighborhood constraints and lead to longer paths. See Figure 1 (b and c) for an example of change of story length with stricter clique size requirement. See Figure 1 (a and c) for an example of change of path length with both stricter clique size and stricter distance thresholds. These two parameters hence essentially map the story-finding problem to one of uncovering clique paths in the underlying induced similarity network between documents.

We use the term “clique chain” to refer to a story along with its surroundings connections of evidence. (In contrast, a story only constitutes the junction points between consecutive cliques.) Another way to characterize them is that a clique chain constitutes many stories. For readability purposes, we automatically associate each document of a clique chain with one of its MeSH terms that has the highest frequency in the abstract of the document.

**Figure 2:**
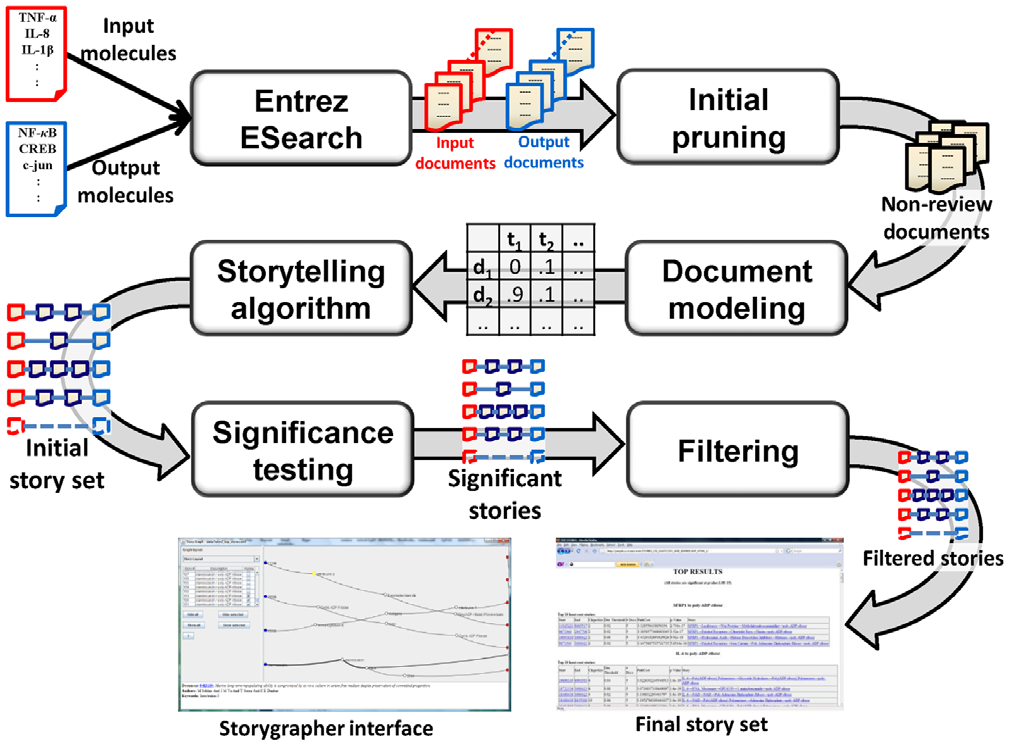
Storytelling pipeline. The pipeline takes a set of input and output molecules, applies algorithmic approaches to handle the research issues in story generation, and at the end outputs significant stories.

We summarize our storytelling framework in Figure 2. The framework takes a set of input and output molecules to relate stories between, applies algorithmic approaches to handle the research issues in story generation, and outputs significant stories at the end of the pipeline. We describe the entire process in detail in the following subsections.

### Identifying Start and End Points for Stories

For each of the case studies, we begin with retrieving, using Entrez ESearch [70] (Figure 2), a large set of documents in PubMed that mention at least one of the molecules from the corresponding list of molecules (Tables 1 and 2 for case studies 1 and 2; PKM2, CTHBP, OIP3, pyruvate kinase, and glutamine for case study 3). We crawl and collect 226,079 PubMed abstracts for the first case study, 444,486 abstracts for case study 2, and 36,100 abstracts for case study 3. Table 3 shows a summary of the three datasets and the seeds used to mine stories.

**Table 3:** Summary of the datasets and storytelling seeds.

	Case Study 1	Case Study 2	Case Study 3
**# of documents by Entrez ESearch**	226,079	444,486	36,100
**# of documents in the final dataset (after initial pruning)**	202,924	291,894	26,536
**# of Terms in the final dataset**	157,209	144,830	55,511
**# of documents labeled with the input molecules**	6,874	895	312
**# of documents labeled with the output molecules**	810	672	228
**# of start-end document pairs**	50,751	5,646	27,251

Some of the molecule descriptions (e.g., ‘serpinB2’) were expanded to cover alternate uses and enhance coverage (e.g., to serpinB2+OR+plasminogen+ activator+inhibitor-2+OR+PAI-2). Of these documents, we eliminate any that have no abstract or title or MeSH terms (this does happen sometimes).

Finally, we remove all review papers from consideration since they provide a ‘lowest common denominator’ approach to storytelling, and lead to simplistic stories involving topic dilution followed by specialization. We provide an example of how a review document can become a ‘lowest common denominator’ and obstruct the specialized flow of a story. Consider first the following story

▪ MMP-9 (PubMed ID: 19556529) → Vascular Endothelial Growth Factor A (PubMed ID: 19073180) → Isoquinolines (PubMed ID: 10350529) → Isoquinolines (PubMed ID: 9334719) → poly-ADP-ribose (PubMed ID: 6458707).

which connects MMP-9 with PARP-1. Silencing/inhibition of MMP-9 and PARP-1 both reduce damage after cerebral ischemia. Since PARP is known to mediate inflammatory responses, it could plausibly modulate MMP-9 activity. As Nampt and MMP-9 activities are known to be linked through NF-kB, such a relationship is highly plausible and warrants further investigation. When review documents are allowed in the storytelling framework, the story above can get “short-circuited” as:

▪ MMP-9 (PubMed ID: 19556529) → PubMed ID: 20410869 → poly-ADP-ribose (PubMed ID: 6458707).

where the only intermediate PubMed article (ID 20410869) is a review paper explaining the ways to repurpose an old drug, minocycline, to improve the use and safety of tissue plasminogen activator for acute ischemic stroke. MMP-9 and PARP-1 are mentioned in the abstract of this review paper but this connection is very superficial and does not provide a detailed insight into the molecular machinery as did the previous story. The propensity of review papers to yield such “surface level” connections motivated us to remove all review papers from consideration.

Although PubMed's metadata tags reviews as such, there are some that slip through this filter, e.g.,:

T. Sugimura and M. Miwa, “Poly(ADP-ribose): historical perspective”, *Molecular and Cellular Biochemistry*, Vol. 138, No. 1–2, pages 5–12, Sep 1994.

We trapped such papers as those whose titles contain ‘review’ or whose abstracts contain the phrase ‘this review’ (however, other usages such as ‘is reviewed’ can be used in legitimate non-review documents).

All these pruning mechanisms are packaged in the “Initial Pruning” step of the storytelling pipeline (Figure 2). Together, these reduced our collection to 202,924 documents for the first case study, 291,894 for case study 2, and 26,536 for case study 3.

For each of the three case studies, we then label a subset of the documents with the given molecules, where the respective molecule is mentioned in either the title or abstract. This was done by projecting ESearch's results for these molecules onto our seed set, and limiting each molecule to have a maximum of (top-ranked) 700 representative documents, for each of the molecules of each case study. For case study 1, a total of 6,874 documents were labeled with the input molecules, taking care to ensure that no document was labeled with more than one molecule, breaking ties arbitrarily. A different 810 documents were labeled with the output molecule.

The lack of overlap among these labeled documents ensures that we do not *a priori* “plant” overlapping or merging story patterns in our results. For case study 2, we had 895 documents labeled with the input molecules and a disjoint set of 672 documents labeled with the output molecules. For case study 3, a total of 312 documents were labeled as the start set and 228 documents were labeled with the output molecule. Then we prepared (start, end) document pairs for each of the case studies. We made sure that every start-end document pair has no overlap of terms to ensure that the discovered hypotheses are not trivial or gathered from the immediate neighborhood of similarities. We prepared 50,751 such start-end document pairs for the first case study, 5,646 document pairs for the second, and 27,251 document pairs for case study 3.

### Document Modeling

As described earlier, we use a bag-of-words (vector) representation for each document (abstract), selecting 157,209 terms from the documents of case study 1, a total of 144,830 terms from case study 2, and 55,511 terms from case study 3 after Porter stemming, and removing stop words, numerals (e.g., measurements), and DNA sequences (e.g., ‘AAAGGT’).

We adopt the weighting scheme for term *t* of document *d* as:
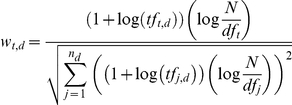
where 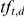 is the frequency of term *t* in document *d*, 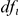 is the number of documents containing term *t*, 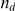 is the number of terms in document *d*, and *N* is the total number of documents. The equation above is a variant of tf-idf modeling with cosine normalization. The PubMed corpora has abstracts of different sizes. In general, longer abstracts have higher term frequencies because many terms are repeated. The cosine normalization helps lessen the impact of size of the abstracts in the modeling.

### Heuristic Search for Stories

Our story searching framework is exploratory in nature so that, given starting and ending documents of interest, it explores candidate documents for path following, and heuristics to admissibly estimate the potential for paths to lead to a desired destination. Our search framework has three key computational stages: (i) construction of a concept lattice using CHARM-L [51], (ii) generating promising successors for path following, and (iii) evaluating candidates for potential to lead to destination. Of these, the first stage can be viewed as a startup cost that can be amortized over multiple path finding tasks. The second and third stages are organized as part of an A* search algorithm that begins with the starting document, uses the concept lattice to identify candidates satisfying the distance and clique size requirements, and evaluates them heuristically for their promise in leading to the end document.

**Figure 3:**
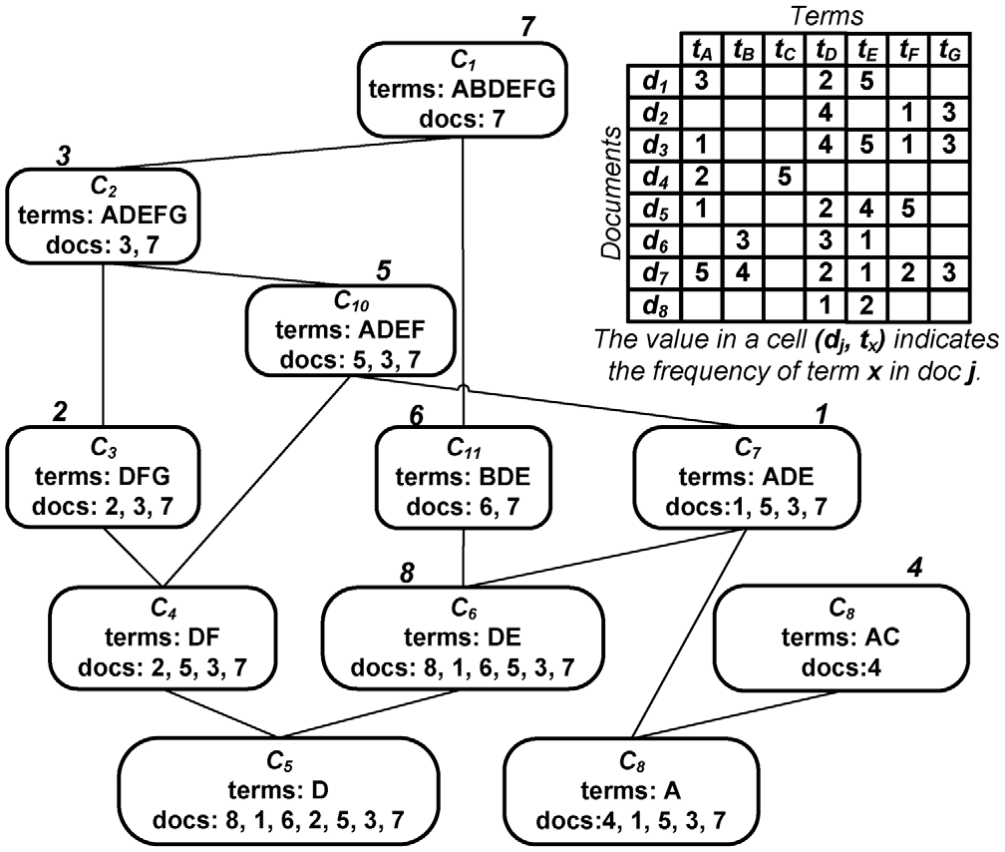
The concept lattice for a given dataset. Each concept is a pair: (document set, term set). We can find an approximate set of nearest neighbors for a document *d* from the document list of the concept containing *d* and the longest term set.

Since our algorithm must satisfy the clique size requirements as well as the distance threshold, we need to efficiently generate a set of good candidate documents from which we can combinatorially form a set of candidate cliques that would potentially force the chain toward the destination. The concept lattice is a data structure that models conceptual clusters of document and term overlaps and is used here as a quick lookup of potential neighbors that will satisfy the distance threshold and clique constraints. Given a (weighted) term-document matrix, we use the CHARM-L [51] closed set mining algorithm on a boolean version of this matrix to generate a concept lattice. Each concept/closed set is a pair: (document set, term set) as shown in Figure 3. Concepts capture maximal co-occurrence between document sets and term sets, i.e., it is not possible to add more documents to a concept without losing some terms, and vice versa.

We order the document list for each concept by the number of terms. In this manner, we can find an approximate set of nearest neighbors for a document *d* from the document list of the concept containing *d* and the longest term set. Successor generation is the task of, given a document, using the distance threshold and clique size requirements to identify a set of possible successors for path following. Note that this does not use the end document in its computation. The basic idea of our successor generation approach is, in addition to finding a good set of successor nodes for a given document *d*, to be able to have sufficient number of them so that, combinatorially, they contribute a desired number of cliques. With a clique size constraint of *k*, it is not sufficient to merely pick the top *k* neighbors of the given document, since the successor generation function expects multiple clique candidates. (Note that, even if we picked the top *k* neighbors, we will still need to subject them to a check to verify that every pair satisfies the distance threshold.) Given that this function expects *b* clique candidates (where *b* is the branching factor), a minimum *m* documents must be identified where *m* is given by the solution to the inequalities:
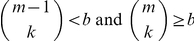
For a given document, we pick the top *m* candidate documents from the concept lattice and form combinations of size *k*. Our successor generator thus forms combinations of size *k* from these *m* documents to obtain a total of *b k*-cliques. Since *m* is calculated using the two inequalities, the total number of such combinations is equal to or slightly greater than *b* (but never less than *b*). Each clique is given an average distance score calculated from the distances of the documents of the clique and the current document *d*. This aids in returning a priority queue of exactly *b* candidate *k*-cliques. We evaluated our successor generation mechanism by comparing it to the brute force nearest neighbor search and the cover tree based [71] nearest neighbor search mechanisms. We found that our concept lattice based successor generation mechanism works more than a hundred times faster than the brute force approach and on an average twenty times faster than the cover tree based approach. Therefore we adopt the concept lattice in our successor generation procedure.

We now have a basket of candidates that are close to the current document and we must determine which of these has the potential to lead to the destination document. The primary criteria of optimality for the A* search procedure of our framework is the cumulative Soergel distance of the path. We use the straight line Soergel distance for the heuristic and, because it obeys the triangle inequality, it can be shown that this will never over estimate the cost of a path from any document *d* to the goal. Therefore our heuristic is admissible and our A* search will yield the optimal path constrained under certain branching factor *b*. We also apply the Soergel distance threshold *θ* that must be satisfied between the vectors that aim to form a clique, or eventually a clique chain. It is important to note that our algorithm never explicitly computes or materializes the underlying network of similarities at any time. As a result, it is very easy to vary the clique size and distance thresholds to analyze different stories for the same start and end pairs.

### Significance Testing

For a story with clique size requirement *k* and distance threshold *θ*, at each step we build a queue of candidate documents by investigating the corresponding concepts of the concept lattice. We apply our heuristic to bring the best candidates at the head of the queue. To calculate the *p*-value, we randomly pick up *k-1* documents from the entire candidate pool and check if all the edges of the formed *k*-clique satisfy the distance threshold *θ*, iterating the test 50,000 times. This allows us to find *p*-values down to 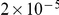. We repeat this process for every junction-document of a discovered clique chain. The overall *p*-value of a clique chain is calculated by multiplying all the *p*-values of every clique of the chain. We compute *q*-values for every clique chain to backup our test using the *p*-values. The *q*-value of a test measures the proportion of false positives incurred (false discovery rate) when that particular test is called significant. We used the qvalue package of Comprehensive R Archive Network (CRAN) to compute the *q*-values for the computed set of *p*-values.

Although intuitively it is easy to realize that stricter thresholds would provide the most significant stories, in practice, for all the case studies we observed that the region between distance thresholds 0.92 to 0.86 and clique size 2 to 8 gives us the most significant stories with low average *p*-values and *q*-values. Most of our final stories fall within these ranges of thresholds.

### Context Overlap Filter

One of the most common problems that prevails in our initial story sets is that the documents in a story do not necessarily stick to one experimental context. For example, a story might meander between different organisms, cell lines, and types of experiments. This can be disorienting to a reader, and it may not even be valid to make a connection between two experimental results that operate on completely unrelated organisms.

To address this problem we first analyzed the structure of a set of PubMed abstracts and determined that most abstracts follow a broad pattern, first stating the background/context of the paper or experiment and then stating the experimental results or conclusions. This implies that sentences closer to the title are more likely to be context sentences. Hence, by imposing a minimum similarity requirement on the context sentences between consecutive documents in a story, we can distinguish between unfocused stories and tightly connected ones.

But how do we disambiguate the context sentences from the results sentences? We noticed that certain words/phrases appear more often in context sentences than in results sentences (e.g., investigated, known to, study, etc.) and vice versa (e.g., evident, identified, significant change, etc.). This implies that the appearance of some words may indicate a higher probability of being either a context or a result sentence. To capture this notion, we design a Naive Bayes Classifier to automatically distinguish between context and results sentences in each abstract. By imposing a minimum Jaccards coefficient between the Context sentences of all consecutive pairs of documents in a given story we can guarantee that there is a minimum consistent context throughout the story.

A Naive Bayes Classifier can be expressed through the equation: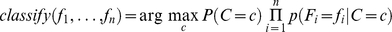.Here *C* is a class variable with a small number of outcomes (Results or Context, a binary variable, in our case), conditional on a number of feature variables *F_1_*, …,*F_n_*. This equation indicates that we can find the best probability of a class given a set of features if we can find the probability of each class occurring and of each feature occurring given that class.

**Figure 4:**
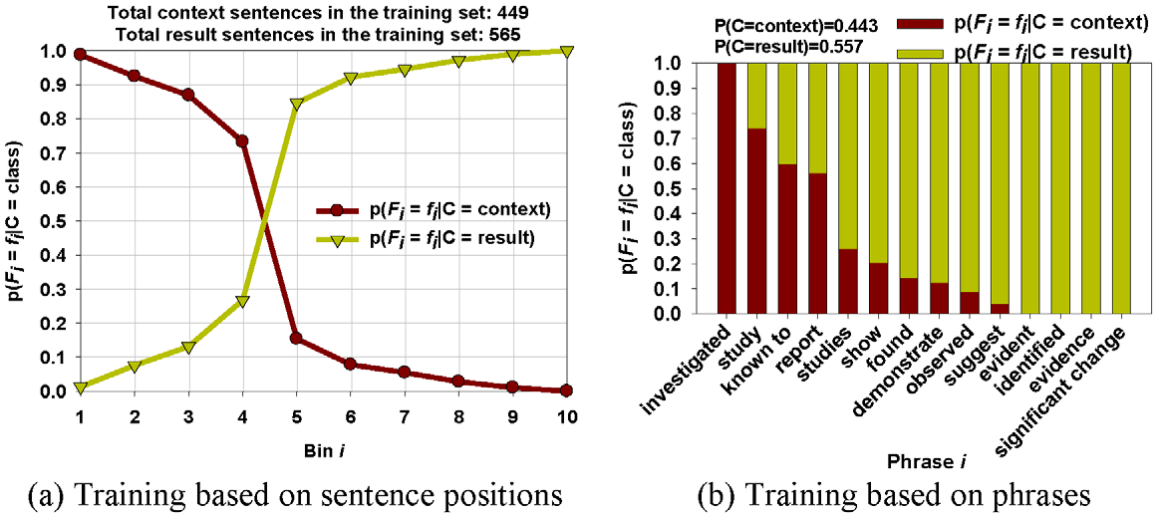
Classification of context/result sentences. The training set contains 100 randomly selected documents (449 context sentences, 565 result sentences) (a) bin probabilities of the training dataset. Sentences closer to the title have higher probability of being a context and furthest sentences from the title tend to be result sentences. (b) phrase probabilities in the training set. Note that some phrases favor a sentence to be a context sentence and some favor it to be a result sentence.

To generate these probabilities we manually analyzed 100 randomly selected documents (from the domain of each case study) as a training set, tagging each sentence as either a Context sentence or Results sentence. These 100 documents had 1014 sentences among which 449 sentences were tagged as context sentences and the other 565 sentences were tagged as result sentences. Context sentences include those related to experimental context, background information, related works, and methodologies. Results sentences include those related to experimental results and conclusions. While tagging each sentence we recorded the Distance From Title (*DFT* = *#preceding/N_s_* where *#preceding* is the number of sentences preceding the given sentence and *N_s_* is the total number of sentences in the abstract). Notice that as a sentence approaches the beginning of an abstract its DFT approaches 0 and as it approaches the end of an abstract its DFT approaches 1. We split the DFT values into 10 equal bins between 0 and 1, tallying the number appearing in each bin for both classes. We then define each bin as a feature *f_i_*, finding probability 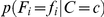 by dividing the number of sentences occurring in that bin for class *c* by the total number of sentences in that bin. Figure 4(a) shows that the Context class has a higher probability near the lower DFT bins and the Results class has a higher probability near the higher DFT bins.

We also marked any words we found that may indicate a strong probability of belonging to specific classes (e.g. ‘investigated’ and ‘study’ appear frequently in Context sentences because they often describe the purpose of the paper or experiment while ‘observed’ and ‘showed’ appear frequently in Results sentences because they tend to report experimental results). We then tally the number of occurrences of each indicator for each class, defining each indicator as a feature *f_i_*, whose probability 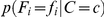 is found by dividing the number of sentences containing that indicator for class *c* by the total number of sentences containing the indicator. Figure 4(b) shows the probabilities of 14 indicator phrases in the training sentences. The probability *P(C = c)* is simply obtained by dividing the number of sentences of class *c* by the total number of sentences in the training set.

**Figure 5:**
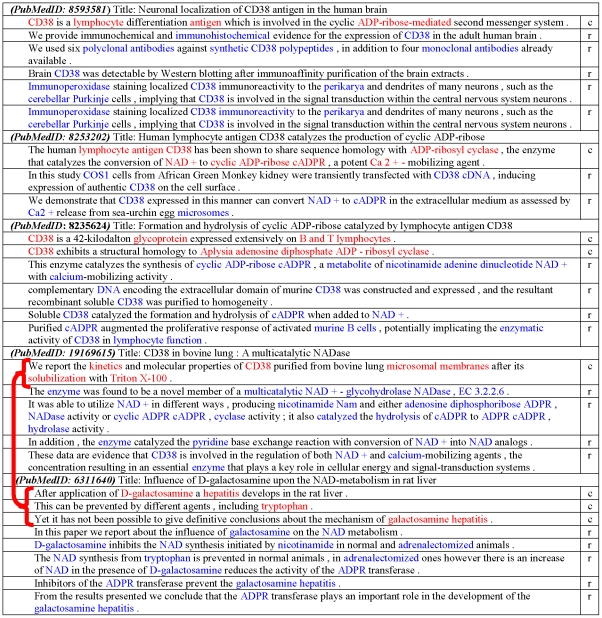
An example of a story with broken context. The story, connecting documents PubMedID: 8593581 and PubMedID: 6311640, is removed by our context overlap filter. The filter recognizes that the last pair of documents do not have any overlap in their context sentences. Each of the sentences of the abstracts of the story is marked by either *c* or *r* to indicate their role as a *context* or *result* sentence. Important terms are highlighted by colors (red is used for the terms in context sentences and blue for the terms in result sentences).

Using the Naive Bayes Classifier equation we predict the sentence classes of each sentence in the abstracts of our stories. We observed an average of 96% accuracy using 10-fold cross validation on the labeled data, which we believe will extend to the remainder of the documents. The 4% discrepancy most likely occurs because some authors report past experimental results (context) without explicitly using words such as ‘previously investigated’. Such sentences require the context of surrounding sentences in order to classify properly, and these cases are beyond the scope of our study. Now we can prune stories that do not have significant context overlap in each consecutive pair of documents. Figure 5 shows an example story that was pruned by our context overlap filter for broken context.

### Sentence Cohesion Filter

**Figure 6:**
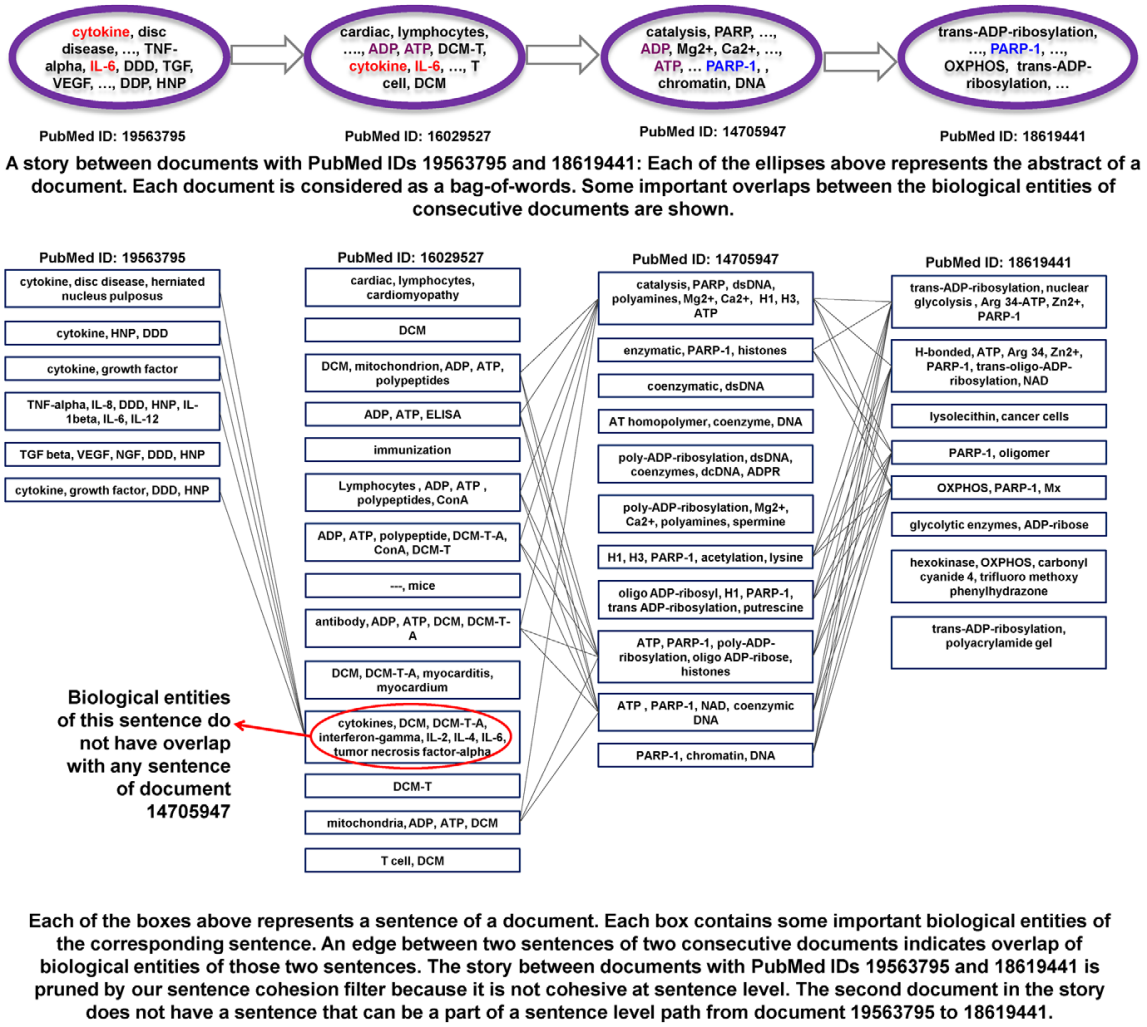
An example story that was pruned by our sentence cohesion filter. The figure shows that a story between PubMedIDs 19563795 and 18619441 is pruned because it does not have a cohesive sentence-level path.

**Figure 7:**
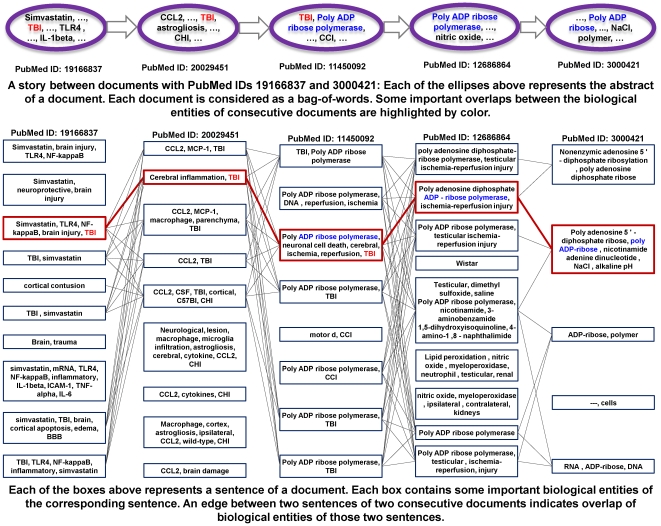
An example story that passes our sentence cohesion filter. The figure shows a story between documents with PubMedIDs 19166837 and 3000421. The story contains many sentence-level cohesive paths from the start document to the end. One of these sentence-level paths is highlighted in this figure.

One consideration in producing coherent stories is whether there exists a progression of sentences from one document to the next that actually relates real biological entities to each other. By analyzing stories at the sentence and biological entity level we can impose restrictions in finer grain, hopefully producing more meaningful stories. With this in mind, we summarize a story by picking key sentences from the abstracts (one from each), and tiling them from start to end documents. We use the LingPipe named entity recognizer [72] to find biological entities in all titles and abstracts (from the original seed set), and create document-sentence and sentence-named entity indices. We then attempt to find paths mirroring the storyline but spanning nodes representing the sentence IDs, and where the Jaccards coefficient is defined by overlap in terms of named entities. All stories that do not have such a mirroring path involving named entities are eliminated (observe that this can happen because the term-document modeling was done prior to named entity extraction). Figure 6 describes an example story that is eliminated by our sentence cohesion filter. Each of the stories that successfully passes the sentence cohesion filter is then summarized by the sentence path going through named entities with the highest (set) Jaccards coefficient between neighboring sets of named entities. Figure 7 shows an example story and depicts the path mirroring the sentence level storyline. Table 4 describes the corresponding story of Figure 7 with the complete and coherent sentences.

**Table 4:** A story between PubMed IDs 19166837 (IL-6) and 3000421 (pADP-ribose).

PubMed ID	Title (followed by the Substance MeSH)	Chosen Sentence
19166837	*(IL-6):* Simvastatin reduces secondary brain injury caused by cortical contusion in rats: possible involvement of TLR4/NF-kappaB pathway.	This study was undertaken to evaluate the effect of simvastatin on the Toll-like receptor 4 (TLR4) and nuclear factor-kappa B (NF-kappaB) related signaling pathway and secondary *brain injury* in rats after *traumatic brain injury (TBI)*.
20029451	*(Chemokine CCL2):*Role of CCL2 (MCP-1) in traumatic brain injury (TBI): evidence from severe TBI patients and CCL2-/- mice.	Cerebral inflammation involves molecular cascades contributing to progressive damage after *traumatic brain injury (TBI)*.
11450092	*(Poly(ADP-ribose) Polymerases):* Traumatic brain injury in mice deficient in poly-ADP(ribose) polymerase: a preliminary report.	*PARP* contributes to neuronal cell death in vivo after cerebral ischemia/reperfusion , however , the role of PARP in the pathogenesis of *traumatic brain injury (TBI)* is unknown.
12686864	*(1,5-dihydroxyisoquinoline):* The effect of poly (adenosine diphosphate-ribose) polymerase inhibitors on biochemical changes in testicular ischemia-reperfusion injury.	*Poly (adenosine diphosphate (ADP)- ribose) polymerase* inhibitors have been used successfully to decrease ischemia-reperfusion *injury* in several organ systems.
3000421	*(poly-ADP-ribose):* Nonenzymic adenosine 5′-diphosphate ribosylation of poly (adenosine diphosphate ribose).	*Poly (adenosine 5′ - diphosphate ribose) (poly (ADP-ribose))* is spontaneously *ADP-ribosylated* when it is incubated with nicotinamide adenine dinucleotide , especially in 0.5 M NaCl and at an alkaline pH.

Figure 1(d) shows the corresponding clique chain.

### Evaluating Stories: Dispersion Plots and the Dispersion Coefficient

**Figure 8:**
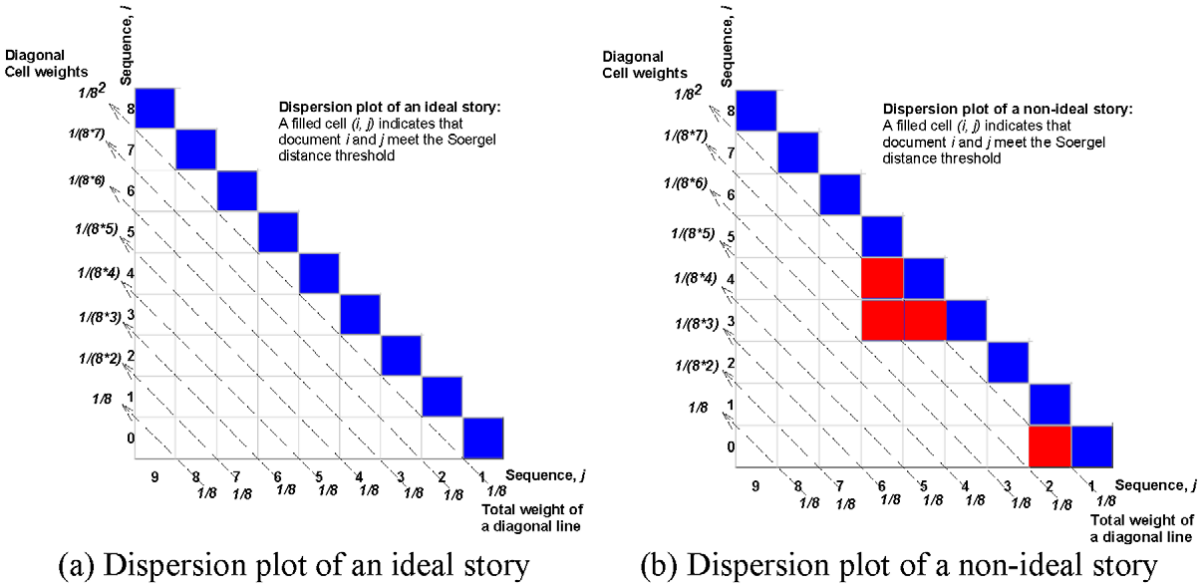
Dispersion plots. The dispersion plot of a story gives an overall idea about overlap of terms between each pair of documents in the story. (a) shows a dispersion plot of a quality story with dispersion coefficient 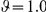, and (b) shows the dispersion plot of a story with dispersion coefficient 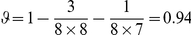.

**Figure 9:**
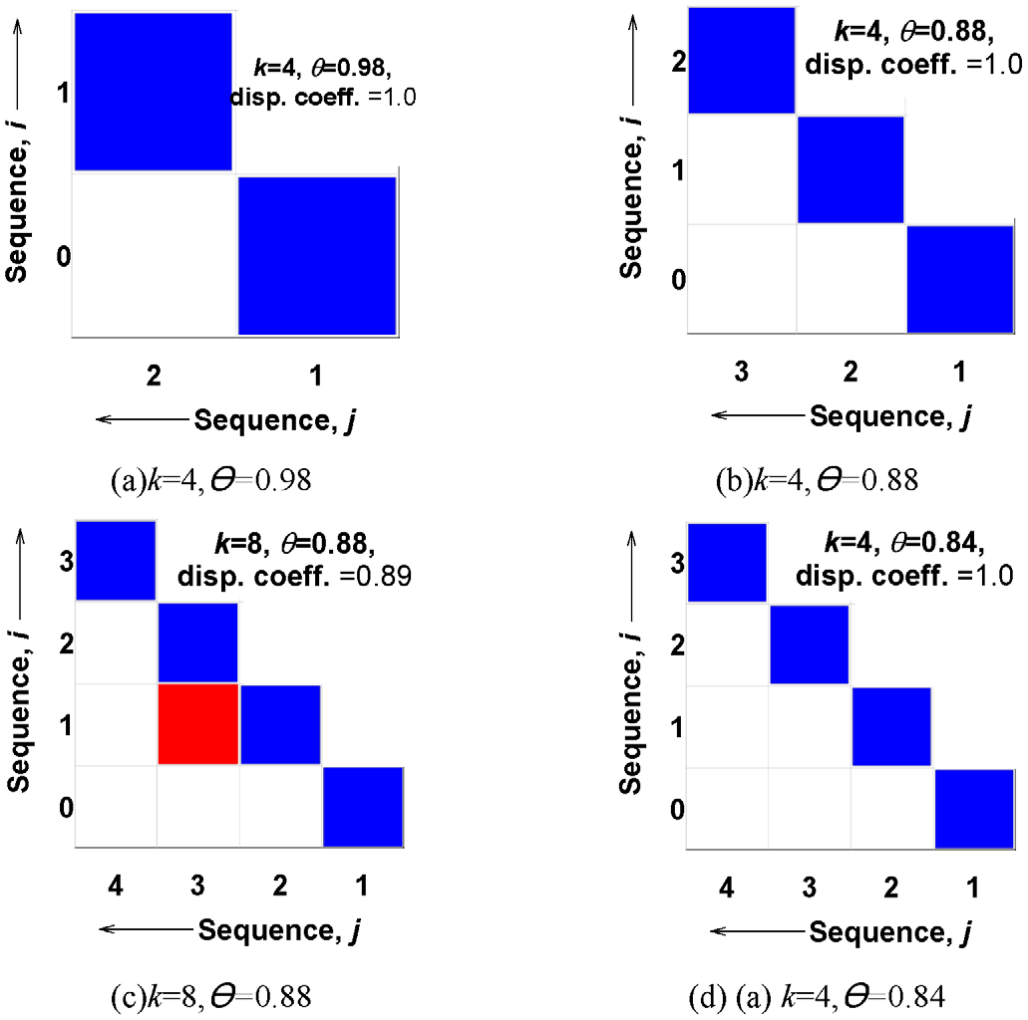
Dispersion plots and dispersion coefficients. Dispersion plots a, b, c, and d of this figure are associated with the respective stories of Figure 1.

For numerical measures of story quality, we adopt Swanson's CBD hypothesis [42] and assess the pairwise Soergel distance between documents in a story, between consecutive as well as non-consecutive documents. We populate the cells of a grid, called a dispersion plot, for which the corresponding pairs of documents satisfy the Soergel distance threshold. Note that a dispersion plot is a way to assess the overlap of the contents of the documents of a story, not the graph structure of the clique chain. (In other words, a dispersion plot evaluates the story, not the clique chain). An ideal story is the one that meets the Soergel distance threshold 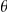 only between consecutive pairs whereas a non-ideal story “oversatisfies” the distance threshold and meets it even between non-consecutive pairs. As shown in Figure 8(a), an ideal story has only diagonal entries in its dispersion plot (contrast with Figure 8(b)). If *n* documents of a story are *d_0_, d_1_, …, d_n-1_*, then we quantify dispersion coefficient as:
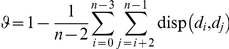
where
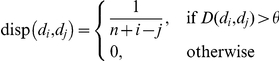
Note that a filled cell near the lower left corner of the dispersion plot penalizes more than a filled cell near the boundary of the plot. Dispersion coefficient is 1 for an ideal story and 0 in the worst case when all the cells of the corresponding dispersion plot are filled. Figure 9 shows four dispersion plots of the stories described by the clique chains of Figure 1. It shows that clique chains of Figure 1 (a, b and d) are ideal and Figure 1 (c) is non-ideal but still possesses high dispersion (0.89). Each of the stories discovered by our framework is associated with a dispersion plot and a specific dispersion coefficient. This helps us to understand the quality of the generated stories in terms of CBD structures.

### Complexity Analysis

The storytelling pipeline has the following stages: pre-processing, indexing, story generation, significance testing, and post-processing. Pre-processing involves stop-word removal, stemming, and document modeling. The document modeling has time complexity 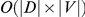 where 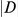 is the number of documents and 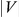 is the size of the vocabulary (i.e., terms). The preprocessing of a dataset of around two hundred thousand PubMed abstracts takes around half an hour on a regular desktop with an Intel Core2 Quad CPU (Q9450@2.66GHz) and 8GB RAM.

We construct a concept lattice using CHARM-L [51] which is used to index possible nearest neighbors. Construction of a concept lattice typically has time complexity 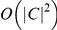 where 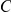 is the number of closed itemsets (concepts). To construct the concept lattice faster, CHARM-L extends CHARM to directly compute the lattice while it generates the closed itemsets. The basic idea is that, when a new closed set 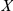 is found, CHARM-L determines all its possible closed supersets. We adopt CHARM-L in the storytelling framework because it is experimentally proven to have faster construction time when compared to some similar techniques [73]. On a dataset of 200 thousand documents and 150 thousand terms, CHARM-L takes several seconds to compute the concept lattice (with minimum support of around 0.0005%).

To keep the pre-processing costs down and the storytelling algorithm runnable on a regular desktop, we find paths in the induced similarity network without materializing the network in its entirety. Therefore generation of one story involves the cost of a regular A* search algorithm plus the cost to find candidate nearest neighbors: 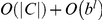. Note that the regular worst case time complexity 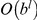 of A* search is actually the complexity of the breadth-first-search algorithm where *b* is the branching factor and *l* is the length of the path. In practice, the complexity of A* search depends on the heuristic *h*. (In case of breadth-first-search, *h* = 0.) We observed that on an average, our heuristic based A* search algorithm is three times faster than the uninformed breadth-first search algorithm. Given a start and end document, discovery of a story generally requires few seconds to a minute depending on the stringency of the distance threshold and clique size parameters.

The significance check of a story has 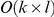 time complexity where *k* is the clique size and *l* is the length of the story. Typically, it requires few seconds to a minute to compute the significance of a story.

Post-processing involves applying the context overlap filter and the sentence cohesion filter. The training of the Naive Bayes Classifier of the context overlap filter has time complexity 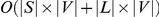 where 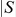 is the number of sentences in the training set, 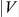 is the length of the vocabulary (the length of the vocabulary is the total number of bins plus the number of words and phrases to identify the result/context sentences), and 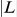 is the number of classes which refers to two classes “context” and “result”. The classification (testing) of a sentence has time complexity 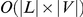. Therefore, both training and testing complexities are linear in the time it takes to scan the data. The training phase takes less than two minutes and a classification requires less than a second on a regular desktop.

The sentence cohesion filter requires path searches from sentences of the start document to the end document. Since the length of a story is comparatively much smaller than the number of documents in the dataset, we use breadth-first-search for the sentence cohesion filter and to build a cohesive sentence level storyline. It requires less than a second to identify a sentence-level storyline or to detect that one does not exist.

Among all the steps described above, the pre-processing and indexing are startup costs that can be amortized over many storytelling runs.

## Results

In this section, we present a comprehensive evaluation of our storytelling framework against a broad range of qualitative and quantitative measures. The specific questions we seek to answer are:

- How are the discovered stories influenced by the distance and clique size thresholds? (Subsection B)
- Do the discovered stories yield domain-specific insight? (Subsection C)
- Can we use the storytelling algorithm to identify emerging areas of study? (Subsection D)
- Do stories discovered by our framework exhibit strong CBD structures? (Subsection E)
- Does storytelling yield qualitatively different insight than traditional cluster analysis? (Subsection F)
- Can storytelling yield fundamentally better connections than mere compositions of PubMed's “related citation” links? (Subsection G)

### A. Summary of the Generated Stories

Table 5 provides statistics about the stories after each important stage of the storytelling pipeline. It shows that initially we obtained 332,104 stories for the first case study, 74,893 stories for case study 2, and 119,964 stories for case study 3. We filter the stories based on statistical significance (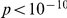, 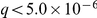) and obtained 17266, 6486, and 11739 stories respectively for case study 1, 2, and 3. For case study 1, we obtain 611 stories after the context overlap filtering and 159 final stories after sentence cohesion filtering. For case study 2, we had 424 stories after applying the context filter and 135 final stories after sentence cohesion filtering. The number of stories after context filtration for case study 3 is 202, while the final set contained 48 stories. The generated stories can be found in the following link of supplementary information: https://bioinformatics.cs.vt.edu/connectingthedots/stories.html

**Table 5:** Summary of number of stories at every filtering step.

# of Stories	Case Study 1	Case Study 2	Case Study 3
After A* search	332,104	74,893	119,964
**After ** ***p*** ** and ** ***q*** **-value filtering** **(** 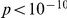 , 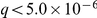 **)**	17,266	6,486	11,739
**After context filtering**	611	424	202
**After Sentence cohesion filtering**	159	135	48

The number of stories reduces as we apply our filters. The final set of stories is statistically significant, contextual, and coherent.

### B. Effect of Distance and Clique Size Thresholds

**Figure 10:**
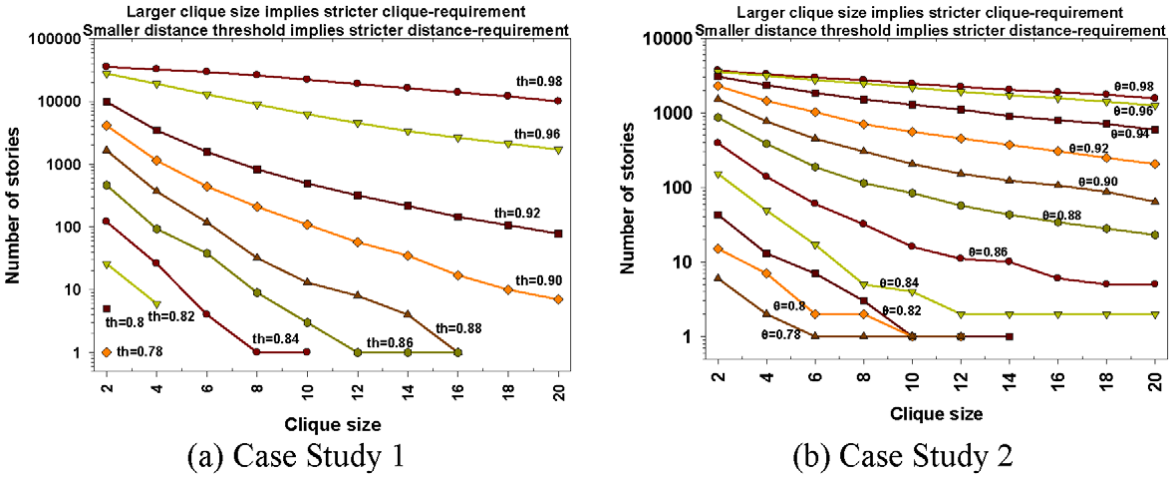
Variation in number of stories. The number of stories generated decreases monotonically with stringent clique size and distance threshold requirements. Plot (a) shows the number of stories with different parameters but same 50,751 start-end document pairs of case study 1. Plot (b) shows a similar graph with 5,646 start-end document pairs of case study 2. Note that both the plots depict monotonic decrease in number of stories with stringent clique size and distance threshold parameters.

**Figure 11:**
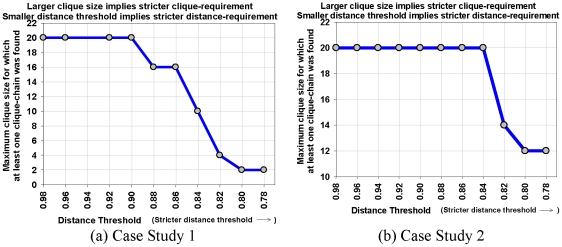
Relation between clique chain and distance threshold. This plot shows the largest *k* for which at least one *k*-clique chain is discovered with different distance thresholds. Note that the stricter the distance threshold is the smaller such *k* is. Both the case studies exhibit the same monotonic relationship between clique size and distance threshold requirements.

**Figure 12:**
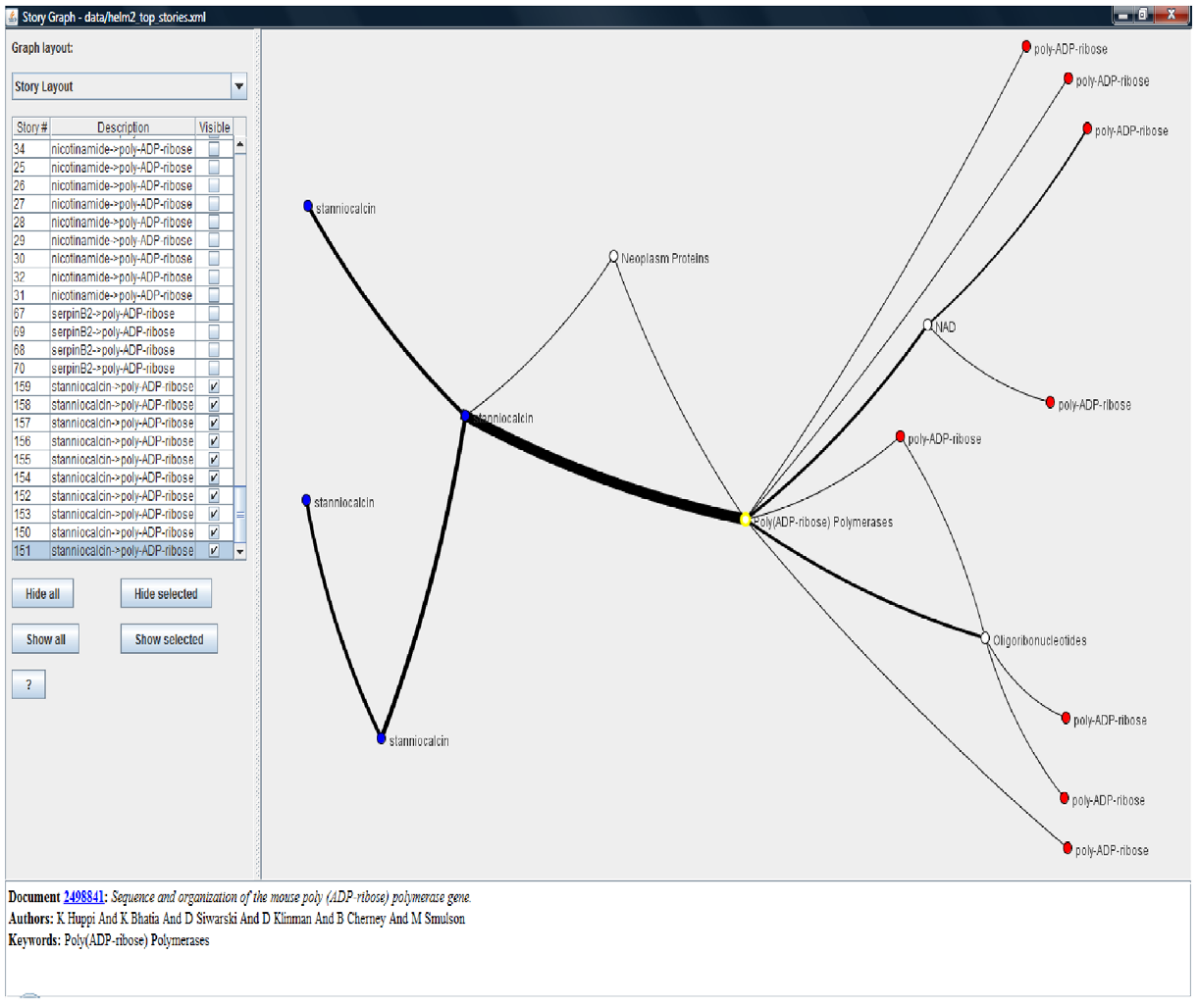
A screenshot of the Storygrapher interface. 10 most significant stories beginning with documents labeled by *stanniocalcin* ending at documents marked by *poly-ADP-ribose are displayed*. Storygrapher helps in analyzing significant stories in a single spatial environment.

**Figure 13:**
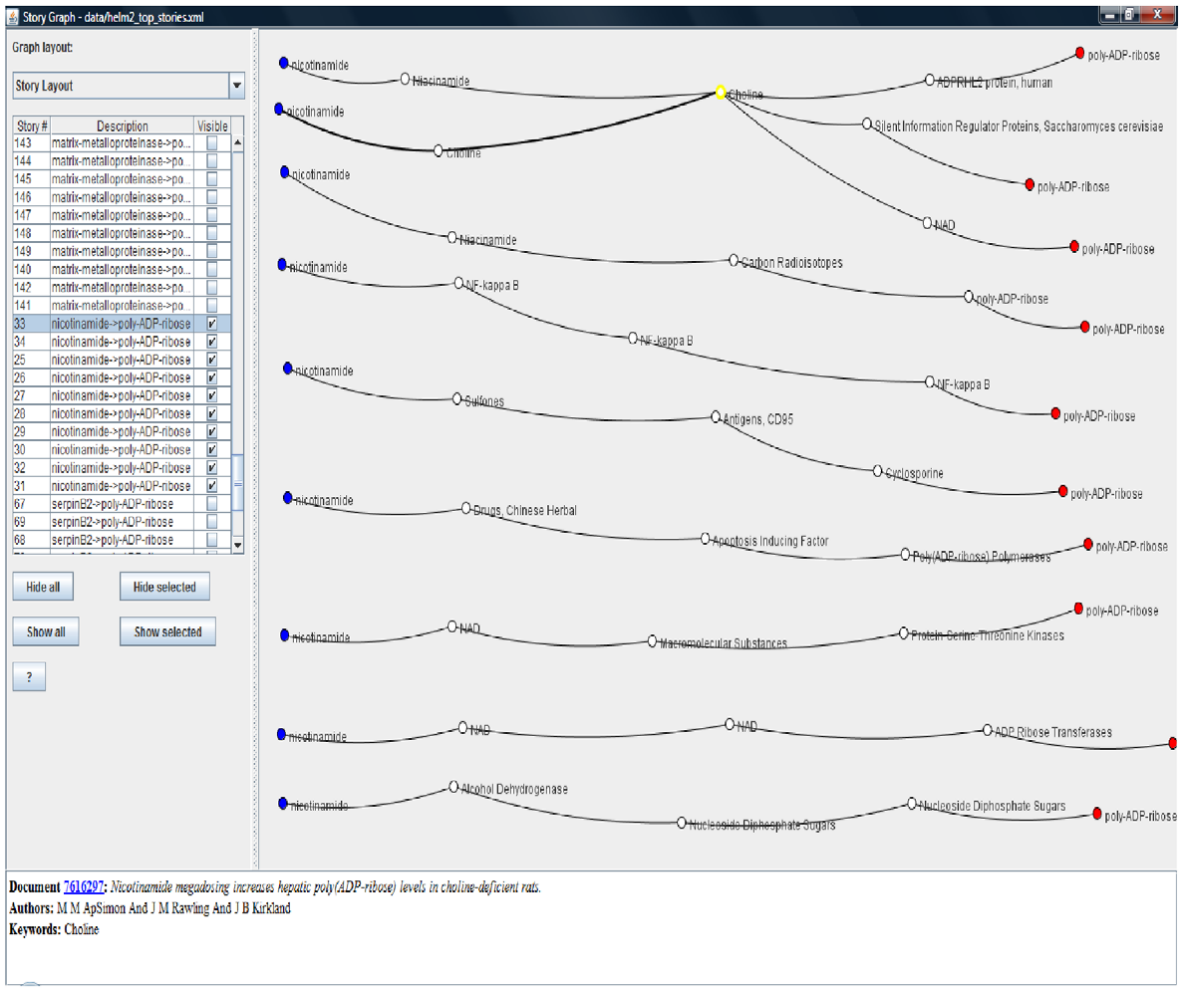
A screenshot of the Storygrapher interface. The screenshot displays 10 most significant stories starting with documents marked by *nicotinamide* ending in documents marked by *poly-ADP-ribose*.

We use case studies 1 and 2 to study the effects of varying the distance and clique size thresholds on the number of discovered stories. The effect of user experimentation with distance and clique thresholds leads to expected results, viz. the number of possible stories decreases monotonically with stricter distance and clique size requirements. Figure 10 reflects conformance to this expectation from our experimental results. Figure 11 shows the relationship between the clique size requirement and distance threshold. For both the case studies considered in this subsection, the largest available clique size becomes smaller with stricter distance threshold. This indicates that the neighborhoods with stronger links are smaller. Figure 12 and Figure 13 show two sample screenshots of the *Storygrapher* interface we have developed for analyzing the resultant stories.

### C. Domain Specific Insight

We describe below domain specific insights extracted by the storytelling algorithm in application to our first two case studies.

#### Case study 1

In case study 1, we explore combinatorial relationships in the literature between a set of 18 input extracellular molecules (see Table 1) and one output, i.e., (poly)ADP-ribose. A total of 159 different stories were discovered by the storytelling algorithm in this case study. We describe few of the discovered stories below.

Table 4 describes the story of Figure 1(d) with coherent sentences. The story begins with document ID 19166837, which was selected as a seed document representing the term IL-6 and ends at a document with ID 3000421 that represents poly-ADP-ribose. IL-6 is one of the inflammatory cytokines released after brain injury, ischemia/reperfusion, etc. and poly-ADP-ribose deficiency or inhibition reduces damage from those inflammatory processes. The story fosters a conceivable hypothesis that poly-ADP-ribose could impact signaling of IL-6 or other interleukins. Table 6 also shows a very similar story between two documents with IDs 18817984 (MCP1) and 17075046 (poly-ADP-ribose). This story connects PAR/PARP to another inflammatory cytokine (MCP1) by way of their respective roles in the MPTP model of Parkinson's disease.

**Table 6:** A story between PubMed IDs 18817984 (MCP1) and 17075046 (poly-ADP-ribose).

PubMed ID	Title (followed by the Substance MeSH)	Chosen Sentence
18817984	*(MCP1):* The effects of MPTP on the activation of microglia/astrocytes and cytokine/chemokine levels in different mice strains.	The effects of *MPTP* on two mouse strains with different *MPTP* sensitivities and immunological backgrounds were compared : *MPTP*-sensitive C57BL/6 *mice* (B6) with a propensity for Th1 and less *MPTP*-sensitive BALB/c mice (BALB) with a propensity for Th2.
9754903	*(1-Methyl-4-phenyl-1,2,3,6 tetrahydropyridine):* Microglial and astrocytic involvement in a murine model of Parkinson's disease induced by 1-methyl-4-phenyl-1,2,3,6-tetrahydropyridine (MPTP).	We have studied the reaction of glial cells in *mice* treated with an intraperitoneal administration of *1-methyl-4-phenyl-1 ,2,3,6 tetrahydropyridine (MPTP)*, a selective neurotoxin of dopaminergic nigrostriatal neurons.
19998477	*(Parp1 protein, mouse):* Poly(ADP-ribose)polymerase inhibitor can attenuate the neuronal death after 1-methyl-4-phenyl-1,2,3,6-tetrahydropyridine-induced neurotoxicity in mice.	Here we investigated the therapeutic effect of the *PARP* inhibitor benzamide against *1-methyl-4-phenyl-1 ,2,3,6 tetrahydropyridine (MPTP) neurotoxicity* in *mice*.
10318960	*(1-Methyl-4-phenyl-1,2,3,6-tetrahydropyridine):* Poly(ADP-ribose) polymerase activation mediates 1-methyl-4-phenyl-1, 2,3,6-tetrahydropyridine (MPTP)-induced parkinsonism.	We show that *mice* lacking the gene for *poly (ADP-ribose) polymerase (PARP)*, which catalyzes the attachment of ADP ribose units from NAD to nuclear proteins after DNA damage , are dramatically spared from MPTP neurotoxicity.
17075046	*(poly-ADP-ribose):* The 39-kDa poly(ADP-ribose) glycohydrolase ARH3 hydrolyzes O-acetyl-ADP-ribose, a product of the Sir2 family of acetyl-histone deacetylases.	ARH3-catalyzed generation of *ADP-ribose* from O-acetyl-*ADP-ribose* was significantly faster than from *poly (ADP-ribose)*.

This story connects two documents, one labeled with an inflammatory cytokine (MCP1) and the other with PAR/PARP. They are connected by way of their respective roles in the MPTP model of Parkinson's disease.

Tables 7 show an example connecting nicotinamide (ID: 12389004) to PAR/PARP (ID: 11420111). It is more interesting when the individual papers in the story are observed, especially in light of NAM lifespan extension. The focus of the intermediate paper with PubMed ID 16701870 is actually Nampt, the rate-limiting enzyme in the NAD salvage pathway [74]. There are intercellular and extracellular forms of Nampt in humans, with the extracellular form linked to insulin resistance and inflammation. Nampt can increase the transcriptional activity of NF-kB, which is known to be dependent on PARP. Considering all these facts, one can build the hypothesis that “PARP activity modulates Nampt”.

**Table 7:** A story between PubMed IDs 12389004 (nicotinamide) and 11420111 (poly-ADP-ribose).

PubMed ID	Title (followed by the Substance MeSH)	Chosen Sentence
12389004	*(nicotinamide):* Pre-B-cell colony-enhancing factor, a novel cytokine of human fetal membranes.	STUDY DESIGN: *PBEF* was immunolocalized in the *fetal membranes* from early pregnancy , at preterm , and at term.
16701870	*(NF-kappa B):* Pre-B-cell colony-enhancing factor (PBEF/Visfatin) gene expression is modulated by NF-kappaB and AP-1 in human amni otic epithelial cells.	These data show that an inflammatory stimulus in the *fetal membranes* inducing *NF-kappaB* and *AP-1* would up-regulate *PBEF* as well as *IL-8*.
12748173	*(NF-kappa B):* Transcriptional regulation of interleukin (IL)-8 by bradykinin in human airway smooth muscle cells involves prostanoid-dependent activation of AP-1 and nuclear factor (NF)-IL-6 and prostanoid-independent activation of NF-kappaB.	Indomethacin , a cyclooxygenase inhibitor , partially inhibited *IL-8* release and the promoter binding of *AP-1* and *NF-IL-6* , but not *NF-kappaB*.
10494847	*(NF-kappa B):* A role of poly (ADP-ribose) polymerase in NF-kappaB transcriptional activation.	Here we show that *poly (ADP ribose) polymerase (PARP)* is required for specific *NF-kappaB* transcriptional activation in vivo.
11420111	*(poly-ADP-ribose):* Characterization of poly(ADP-ribose)polymerase from Crithidia fasciculata: enzyme inhibition by beta-lapachone.	Crithidia fasciculata *poly (ADP-ribose) polymerase (PARP)* has been isolated and partially purified.

This story connects nicotinamide to PAR/PARP and is more interesting when the individual papers in the story are observed, especially in light of NAM lifespan extension. The focus of the paper with PubMed ID 16701870 is actually Nampt, the rate-limiting enzyme in the NAD salvage pathway. This enzyme also acts as a cytokine and is modulated by NF-kB activity, which is known to be dependent on PARP. Considering all these facts, one can build a hypothesis that PARP modulates Nampt.

To the best of our knowledge, although some papers have explored their underlying relationships [75], [76] there is no single publication that has directly conducted an experiment to assess PAR/PARP effect on IL-1 activity. Our framework generates a hypothesis that treatment with IL-1 alpha and PARP inhibitors would attenuate post-burn ischemia/reperfusion injury and intestinal permeability. The PubMed document IDs in this story are: 12496536→ 20009662→15555790→12626333→8080500.

Our framework sometimes generates stories which may warrant further investigation rather than directly composing a hypothesis. An example of such a story is: 19556529→ 19073180→10350529→9334719→6458707. This story connects MMP-9 with PARP-1. Silencing/inhibition of MMP-9 and PARP-1 both reduce damage after cerebral ischemia. Since PARP is known to mediate inflammatory responses, it could plausibly modulate MMP-9 activity. As Nampt and MMP-9 activities are known to be linked through NF-kB, such a relationship is highly plausible.

#### Case study 2

The stories generated in case study 2 are based on multiple cytokine/chemokine inputs and multiple transcription factor outputs (Table 2). Three of the four inputs are inflammatory cytokines whereas IFN-gamma is typically associated with innate and/or adaptive immunity. The paths from cytokine to transcription factor are well worn in the research community, and several of inputs are well established as being linked to the outputs used. However, many of the established signaling pathways are cell type specific and/or are present only aberrant disease states such as cancer. Thus prior to the analyses one could expect relatively short and statistically significant stories relative to the “many in, one out” case study as long as stories remain mapped to specific cell types or lines. One can also envision paths that are more complex and difficult to interpret due to the pleiotropic behavior of signaling cascades, which could be exacerbated if multiple cell lines or types are pulled into a story. Graded responses, where relative amounts of individual inputs and outputs control cellular fate, gene mutations and splice variants also present challenges in such a search.

A total of 135 different stories were discovered by the storytelling algorithm. Several were selected at random for further evaluation, of which two are described here. Amyotrophic Lateral Sclerosis (ALS, Lou Gerhig's Disease) is a fatal motor neuron disease with no present cure. A subset of ALS patients have mutations in the gene encoding for 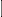
Cu-Zn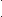 superoxide dismutase (SOD1), an enzyme that converts toxic superoxide to hydrogen peroxide and oxygen. The aberrant enzyme can accumulate as fibrillar aggregates creating a cytoxic state and an inflammatory response. In the story relating IL-1 beta to JunD: 20616033→19733170→1373742, we start with a recent finding on the connection between the SOD1-induced inflammation and a treatment to reduce the IL-1beta response (PubMed ID: 20616033). The ALS connection was carried into the next publication (PubMed ID: 19733170), a study that demonstrated that the administration of macrophage colony stimulating factor (M-CSF, a pro-survival cytokine) accelerated disease progression, an outcome suggested to be due to the upregulation of pro-inflammatory cytokines such as IL-1beta. The connection between M-CSF and ALS and the transcription factors junB and c-fos in a myeloid leukemia cell (PubMed ID: 1373742) line was made through M-CSF. While junB was not in our original output list of transcription factors, c-fos, c-jun, junB, and junD were all evaluated in this study. It was found that junB and c-fos expression was elicited when M-CSF was administered, but c-jun and junD were not. There may be a connection between junB, c-fos and ALS.

The story line: TNF-alpha (PubMed ID: 20108015) →NF-kappa B (PubMed ID: 9779924) →NF-kappa B (PubMed ID: 2691328) →Tumor Necrosis Factor-alpha (PubMed ID: 19706766) →JunD (PubMed ID: 11526502) starts with rheumatoid arthritis (an inflammatory response) and the presence of inclusion body myositis (IBM), an inflammatory muscle disease. This condition has been linked to the NF-kB transcription factor, a protein complex whose function is dependent on the presence or absence of specific binding partners. NF-kappaB activation prevents cell death and can be activated by forming a complex with the oncoprotein MUC1-C, permitting cell transformation. Inhibiting MUC1-C decreases the activity of NF-kB, preventing cell transformation, a potential anti-cancer strategy. The final article in the story describes the cooperation of NF-kappaB and JunD in rat liver cell regeneration (a proliferative state). Such processes can be induced by TNF-alpha, a known activator of NF-kappaB. The story line here describes a connection that is already established.

### D. Validation using Warburg Effect

To validate our framework, we perform a date-constrained exploration of documents for the third case study to assess if the storytelling algorithm can identify emerging areas of study. Recall that in this case study, we aim to unravel the relationship between pyruvate kinase and glutamine to assess the ability to recover indicators of the Warburg effect from past literature. Since several key papers related to Warburg effect providing key insights into the metabolic needs of cancer cells appeared in 2008 and 2009 [62], [63], [65], [68], the storytelling algorithm was employed using a set of 36,100 abstracts published *before* 2008. The seed terms to collect this dataset were: pyruvate kinase, PKM2, CTHBP, OIP3, and glutamine. We used pyruvate kinase and glutamine as both start and end points in order to determine if the algorithm can identify a relationship between the two. Table 5 shows the number of stories at different stage of the storytelling pipeline. The final set contains 48 stories. From this final set, a subset of stories that contained the keyword “cancer” was evaluated in an effort to see if the stories generated matched with the findings published between 2008 and the present. There were more stories with cancer in the summary texts when starting with pyruvate kinase (13 stories) than with glutamine (3 stories). All these 16 stories are highlighted by yellow background in the list of stories of case study 3 of the supplementary information website. Two of the three glutamine-initiated stories shared the same path differing only in the starting document:

- glutamine (PubMed ID: 15941893) → Glutamine (PubMed ID: 15990633) → Glutamine (PubMed ID: 12600870) → Glucagon (PubMed ID: 6142902) → Amino Acids (PubMed ID: 2913952) → Glutamine (PubMed ID: 6599917) → pyruvate kinase (PubMed ID: 3732223).
- glutamine (PubMed ID: 17499398) → Glutamine (PubMed ID: 15990633) → Glutamine (PubMed ID: 12600870) → Glucagon (PubMed ID: 6142902) → Amino Acids (PubMed ID: 2913952) → Glutamine(PubMed ID: 6599917) → pyruvate kinase (PubMed ID: 3732223).

Interestingly, both of these stories follow a path of the positive effects of infant glutamine supplemental feeding to the mechanistic aspects of PKM2 function (metabolic programming for growth). As both infants and tumor cells share a similar need for growth, such a connection is consistent with data available after 2008 on the relationship between glutamine, pyruvate kinase and cancer [66], [67].

When starting with the pyruvate kinase term and limiting the analysis to stories containing the term “cancer”, all of the 13 identified stories began with work that described pyruvate kinase as a cancer biomarker. While this clearly shows that PKM2 was an established biomarker for cancer prior to 2008, there was no mention of fetal tissues and/or infants in any of these stories. In addition, the ending glutamine citations focused less on the nutritional aspects of the amino acid (as was the case when it was the starting term) and more on its protective effects against oxidative and heavy metal derived stresses. Clearly the direction at which our storytelling algorithm starts will influence the results obtained; a reflection of the emphasis placed on these two materials (one an enzyme the other a metabolite) as subjects of investigation. No mention was made in any highlighted study as to the role of lactic acid and/or the Warburg effect, and only two stories included a citation related to phosphorylation (9409743) and cell proliferation (18060527):

- pyruvate kinase (PubMed ID: 17632316) → pyruvate kinase (PubMed ID: 15162541) → glucose (PubMed ID: 9409743) → glutamine (PubMed ID: 18060527).
- pyruvate kinase (PubMed ID: 17577247) → pyruvate kinase (PubMed ID: 11326648) → pyruvate kinase (PubMed ID: 15162541) → glucose (PubMed ID: 9409743) → glutamine (18060527).

We interpret these results to indicate that a clear connection between glutamine and pyruvate kinase had yet to be established. With respect to lactate levels, we believe this is due to the commonly held belief that all lactate was derived from pyruvate, not from a more indirect route involving glutamine. In regards to post-translational modifications such as phosphorylation, the role of these modifications was only beginning to be appreciated.

In summary, using the terms pyruvate kinase and glutamine with abstracts limited to pre-2008, supporting evidence was available that could link these two terms together, supporting the concept that the Warburg effect is related to both pyruvate kinase and glutamine.

### E. CBD Structures in Discovered Stories

**Figure 14:**
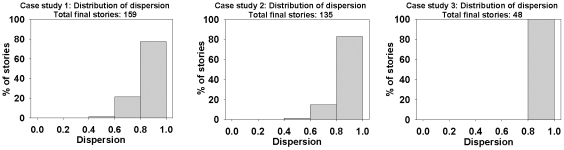
Distributions of dispersion. The plots show the distributions of dispersion of the final set of stories for three case studies, The distributions show that the resulting stories of all the case studies have overall high dispersion coefficient.

As with actual published literary works, there are good stories and not so good ones, and what is considered good is somewhat subjective. Inasmuch as it is somewhat difficult to provide a measure of story effectiveness, as described in the *Methods* Section, for numerical measures of story quality, we adopt Swanson's CBD hypothesis [42] and use dispersion coefficient to quantify the quality of a story. The dispersion coefficient is 1 for an ideal story where only consecutive documents satisfy the Soergel distance threshold and 0 in the worst case when all the pairs of documents in a story satisfy the threshold. Although we do not directly optimize over the dispersion coefficient, the stories discovered by our framework have high dispersion coefficient due to the experimental settings and rigorous filtration process. The smallest dispersion observed was 0.5 in case studies 1 and 2. The smallest dispersion we observed in case study 3 was 0.88. Note that none of the final sets of stories had dispersion coefficient smaller than 0.5. Only around 20% of the stories of case studies 1 and 2 have dispersion coefficient ranging from 0.5 to 0.8. Figure 14 shows the distributions of dispersion of all final sets of stories of all the case studies. The plots depict that around 80% stories of case study 1 and 2, and 100% stories of case study 3 have dispersion coefficient higher than 0.8. High dispersion coefficients in the final sets of stories indicate high quality adoption of Swanson's CBD hypothesis.

### F. Storytelling vs Cluster Analysis

The concept of storytelling is fundamentally different from the document clustering concept. Clustering brings together similar documents whereas stories connect dissimilar objects. The former helps organize a document collection into regions of interest, and the latter explores latent information by connecting the dots between disjoint instances. Depending on its end points, a story might be situated within a cluster in its entirety or might straddle multiple clusters.

**Figure 15:**
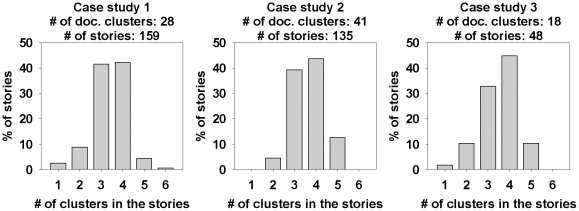
Distribution of clusters in the stories. Each of the plots shows the number of traditional clusters in the stories. The peak is found at four clusters in each of the case studies. The plots show that in each of the case studies, more than 40% of the stories pass through four clusters.

To understand the behavior of our discovered stories from a clustering perspective, we cluster the documents of each of the case studies independently using the *k*-means clustering algorithm. We clustered the documents for each dataset with different numbers of clusters and selected the best number of clusters based on the highest average silhouette coefficient [77]. We found that the best numbers of clusters for case studies 1, 2, and 3 are respectively 28, 41, and 18. After we have the clusters for a case study, we count the number of clusters each of the stories of that case study passes through. Figure 15 shows three plots illustrating percentage of stories passing through different numbers of clusters for three case studies. The distributions are skewed at right with a peak at four clusters. More than 40% stories of each case study pass through four clusters. We observe that most of the stories of all the case studies are from multiple clusters. None of the final set of stories of case study 2 is solely from one cluster (Figure 15 (middle)). The distributions of Figure 15 vividly illustrate that our storytelling framework is able and aimed to entwine documents from different clusters to weave a story.

### G. Storytelling vs Related Citations Exploration

PubMed provides a list of related citations for every document. PubMed's related citations are similar documents which are pre-computed for every article and presented to the user on demand. PubMed computes similarity between two documents based on local and global weights of terms in the documents and involved MeSH terms with a subheading qualifier. It is thus possible to traverse the PubMed citation graph by successively clicking on the “related citations”. In this evaluation, we aim to characterize the differences between traversal of PubMed's related citations graph and the application of our storytelling algorithm.

To inspect whether it is possible to find the discovered stories via the related literature network of PubMed, we collect lists of related citations of all the documents of all the stories discovered by our storytelling framework. For each story, we check if the next document in the story is, in fact, an article from the related citation of the current document. A story is considered to be “found” in the related citation network if all the immediate following documents of the story can be found from the corresponding related citations of the current documents. Table 8 shows the number and percentage of stories that could be found by successively clicking on the related citations in the pubMed website. The table shows the statistics of such stories both before and after any filtration. We observed that respectively 0.02%, 0%, and 0.0008% of the unfiltered stories of case studies 1, 2 and 3 were found in the related citation network. None of the final stories of any of the case studies was found in the related citation network. This indicates that the storytelling framework realizes connections that the related citation structure of PubMed is not able to discover.

**Table 8:** Number of stories that could be found by traversing the related citations of PubMed.

	Case Study 1	Case Study 2	Case Study 3
**Before any filtration**	66 stories (0.02%)	0 stories (0%)	1 story (0.0008%)
**After all the filtrations**	0 stories (0%)	0 stories (0%)	0 stories (0%)

Only a small number of unfiltered stories could be found by exploring related citations provided by PubMed. None of the final set of stories could be discovered by traversal of PubMed's related citations.

## Discussion

We have described a framework that generates hypotheses from PubMed abstracts using a storytelling algorithm. Our framework is exploratory in nature and enables the user to adjust thresholds on distance and clique size to obtain stronger/broader stories. Stories discovered here support more rapid knowledge discovery than simple search engine results: they provide a significant level of data reduction so that the biologist needs to peruse only a small handful of connections from a massive haystack of documents.

Researchers generally enter into a study with a hypothesis to be tested or a question to be answered. Inherent in the endeavor is a bias based the interests of the research team, the goals of the project, and the literature the group tends to focus on. When one factors in the skill sets and resources of the laboratory, there is a limited “experimental space” in which the team can work in. For example, if a biologist is provided a list of regulated genes from the organism under investigation in his/her laboratory, the eyes and thoughts of the researcher will be first directed to the genes that he/she knows something about, not the hypothetical genes or the genes that the researcher has no experience with. Conclusions will typically be based exclusively on what is known by the investigator. The storytelling framework presented here attempts to avoid bias by asking if links can be made between two separate terms that the investigator thinks are related (directly or indirectly). Such a search can provide new insights beyond established pathways, potentially suggesting new experiments. Hence the strength of our storytelling approach is in searching for “what may be” by searching “what is”.

We envisage several directions of future work. First, we aim to develop probabilistic models of story construction based on content similarity (akin to [78]) or based on incremental updation of topic models [79]. Such a model can enable us to do knowledge transfer across storytelling scenarios and build upon prior knowledge systematically to construct more complex stories. Second, we plan to implement a rich model of user interaction by which users can steer the story construction process using examples of desirable and “to-be-avoided” stories (e.g., *apriori* known connections). Such feedback can also be incorporated into the probabilistic modeling framework.

Third, the idea of storytelling to string together documents can be viewed as a form of “compositional data mining” [80] and can be generalized to more forms of biological data than merely published abstracts. Compositional data mining (CDM) is the idea of “chaining” patterns across diverse data sources so that connections can be inferred across related, but only indirectly related, datasets. We have previously shown [81] how, in analyzing *C. elegans* functional genomics datasets, CDM was used to suggest a connection between the Wnt signaling pathway and insulin signaling, mediated by the tyrosine kinase receptor DAF-2, which is involved in longevity. By integrating the capabilities of storytelling presented here with CDM's abilities to mine relational data, we can mine the combined landscape of public domain datasets and publications.

Finally, the care we have taken to discover relevant stories through the design of context filters suggests to us that a broader, community-oriented, emphasis on modeling of biological literature can be useful. Just like minimum information (MI) guidelines exist for different forms of data (e.g., microarray experimental results, SBML models) we can envisage a minimum information about a published article (MIPA) that can help conduct most of the context modeling that we have performed here, and thus provide an impetus to stronger document modeling projects. In fact, some of the methods presented here can be used to impute structure to existing documents, e.g., to partition a document into a “structured abstract” (background + methodology + results + conclusion), and to infer a typology of organisms, stresses, cell lines, and diseases discussed in a given article. Such a typology can then be used for comparison with other documents using semantic measures of similarity [82].
